# A Treatise on Diet, Disordered State of the Digestive Organs, &c.

**Published:** 1827-01

**Authors:** 


### MEDICO-CHIRURGICAL

### REVIEW.

VOL. X.] Analytical Series. [No. 27.

" Nec tibi quid liceat sed quid fecisse decebit
" Occurrat, mentemque domat respectus honesti."

VOL. VI.] JANUARY 1, 1827. [No. 11.

[NEW SERIES.]

### I.

*A Treatise on Diet: with a view to establish, on practical Grounds, a System of Rules for the Prevention and Cure of the Diseases incident to a Disordered State of the Digestive Functions.* By J. A. PARIS, M.D. F.R.S. Fellow of the Royal College of Physicians, &c. &c. One vol. 8vo. pp. 307, closely printed. London, Underwood and Co. 1826.

" Some physiologists will have it that the stomach is a mill;—others, that it is a fermenting vat;—others, again, that it is a stew-pan;—but in my view of the matter, it is neither a mill, a fermenting vat, nor a stew-pan—but a stomach, Gentlemen, a stomach."—*Manuscript Note from Hunter's Lectures.*

THE labours of Dr. Paris are well known, and justly appreciated. During the last thirteen years he has published three important works. The first, or Pharmacologia, was novel in its kind and happy in its execution, and the number of editions which it has reached renders praise useless and censure pointless. The second, or on Medical Jurisprudence, has not received the patronage that it deserved, and this may be attributed to its having been a joint production, and to certain obvious partialities and imperfections. It is, in truth, the least perfect, and most objectionable, of all this author's performances; being too little to comprise the whole of the materials for a complete work, and too large for common and practical purposes. In many respects, indeed, the production is faulty, and loaded with vapid and uninteresting matter; yet still it is ingenious, learned, and scientific; enlarged in its views, comprehensive in its details, and acute in its conclusions; and decidedly without an equal in this, or in any other language. The third is that which is now before us, embracing the consideration of a subject indispensable to the human race, and expressing observations on the prevention, management, and removal of a disease from which few escape; and which, when rivetted in inveteracy, makes rank useless, riches valueless, and existence burthensome. Dr. Paris evidently has a taste, as well as talent, for chemico-medical and popular pursuits; and being, by nature and education, shrewd, penetrating, and industrious, his labours are generally successful, since they exhibit the stamp of a cautious and philosophic mind, which never disregards the dictates of common sense.

It is our intention to analyze the present work at considerable length, for it is written on matters which, in the language of Bacon, "come home to men's business and bosoms." Strong as is our desire to convey valuable information, yet we cannot presume to attempt impossibilities;—we can only do our best, since inclination and design are always imperiously controlled by power and space; and even if our ability of compression were nearly miraculous, we could not hope to comprise within forty pages, that matter, which, in its original shape, occupies three hundred and seven. All we can do is to imitate the epicure, who, because he cannot partake of every dish, and of every wine, at a sumptuous table, contents himself with selecting the rarest and finest. The text shall be devoted to the opinions and doctrines of Dr. Paris; and our own will be expressed, *first*, in the form of notes, and, *secondly*, at the conclusion of the analysis. This will be more just to the author, more lucid to the reader, and more convenient to ourselves.

The Treatise of Dr. Paris consists of three parts:—I. Anatomical View of the Alimentary Canal, also of its various glands and vessels for nutrition—the lungs, kidneys, and skin; the physiological history of digestion; the relations of the digestive functions with our sensations—hunger, thirst, and instinctive desire for exercise. II. The Materia Alimentaria, containing every thing appertaining to animal and vegetable food, cookery, condiments, drinks, fermented liquors, wines, milk, fish, birds, different varieties of bread, pulses, periods for meals, quantity of food, &c. III. Of Indigestion. Imperfect chymification; imperfect digestion in the duodenum. Headachs. Biliary derangement. A tabular scheme, and commentary on it. Cure of indigestion. Practical rules for dyspeptic patients. Acidity of stomach, flatulence, &c. Recapitulation; diet for patients suffering under tabes mesenterica, &c.; conclusion, and illustrative cases.

Correct and well executed as the first part undoubtedly is, we must take leave to pass it over. The anatomy and physiology of the digestive, as well as of the collateral organs, ought to be already well known by every medical reader, and, to the general reader, such knowledge is worse than useless—consequently, it is on the second and third parts of the work, beyond all doubt the most useful and interesting, that our attention and labours shall be concentrated.

### Part II.—*Materia Alimentaria.*

Chemistry has demonstrated the nature of those proximate principles of organic matter, upon the presence of which the nutritive qualities depend, viz. *fibrin, albumen, gelatin, oil and fat, gluten, fecula, or starch, mucilage, sugar, acids, &c.* Assuming that the variety observable in the nutritive value of different substances arises from the predominance of one or more of such principles, the *nutrientia* may conveniently be distributed into the following nine classes:

Cl. I.—*Fibrinous Aliments.* Comprehending the flesh and blood of various animals, especially such as have reached puberty: venison, beef, mutton, hare.

Cl. II.—*Albuminous.* Eggs; certain animal matter.

Cl. III.—*Gelatinous Aliments.* The flesh of young animals: veal, chickens, calf's foot, certain fish.

Cl. IV.—*Fatty and Oily Aliments.* Animal fats, oils, and butter; cocoa, &c.; ducks, pork, geese, eels, &c.

Cl. V.—*Caseous Aliments.* The different kinds of milk, cheese, &c.

Cl. VI.—*Farinaceous Aliments.* Wheat, barley, oats, rice, rye, potatoe; sago, arrow-root, &c.

Cl. VII.—*Mucilaginous Aliments.* Carrots, turnips, asparagus, cabbage, &c.

Cl. VIII.—*Sweet Aliments.* The different kinds of sugar, figs, dates, &c.; carrots.

Cl. IX.—*Acidulous Aliments.* Oranges, apples, and other acescent fruits.

To these, *Condiments* may be added; such as salt, the varieties of pepper, mustard, horse-radish, vinegar, &c.

In the classification of the different species of potations, we may, in like manner, be governed by their distinguishing chemical composition. They may be arranged under four divisions, viz.

CL. I.—*Water*. Spring, river, well-water, &c.

CL. II.—*The Juices and Infusions of Vegetables and Animals*. Whey, tea, coffee, &c.

CL. III.—*Fermented Liquors*. Wine, beer, &c.

CL. IV.—*The Alcoholic Liquors, or Spirits*. Alcohol, brandy, rum, &c.

The terms digestible and nutritive must not be considered as synonimous. A substance may be highly nutritive, and yet digestible with difficulty; for it may require all the powers of the digestive organs to convert it into chyle; but still it may afford a highly restorative principle. So it is with some of the fatty and oily aliments; and it has been calculated that an ounce of fat meat is equal in nutriment to four of lean. Again, there are substances, apparently easily digested, which yield comparatively little support to the frame.

A healthy stomach readily and effectually disposes of solid food of a certain specific degree of density, and this may be termed its *digestive texture*. If the food exceed this, it will require a longer period, and more active powers, to complete its chymification. The same will occur when the food approaches too nearly to a gelatinous condition. It is, perhaps, not possible to appreciate the exact degree of firmness, which will confer the highest order of digestibility upon food. The powers of the stomach may likewise vary in different individuals.* But experience teaches that no meat is so digestible as tender mutton, and it is, in this country, more used than any other kind of animal food. Wedder mutton, or the flesh of the castrated animal, is in perfection at five years, and is indisputably the sweetest and most digestible. Ewe mutton is best at two years' old. Beef is not so easy of digestion, yet, though its texture is firmer, it is equally nutritive. Much, however, will depend upon the time which has elapsed since the

* Much is also to be attributed to what may be termed the *education* of the stomach. The children of peasants feed on fat bacon, and use hog's lard for butter, with ease and pleasure; while these coarse aliments would sicken, and even vomit, those of a higher rank in life. Again, the offspring of the natives of the Polar regions suck pieces of blubber with delight, and their parents eat tallow candles, and drink oil, with rapture. Now all this, we conceive, is owing to the *customs* of barbarous life, as the reverse is to those of civilized society, and not to any natural difference in the powers of the stomach, which are probably, with certain necessary exceptions, the same in the whole of the human race.—*Rev.*

death of the animal, and still more upon the method of cookery. It is easy to understand why a certain texture and coherence of the aliment should confer upon it either digestibility, or the contrary. The conversion of it into chyme is effected by the solvent power of the gastric juice, assisted by the *churning* which it undergoes by the motions of the stomach; and, if the substance does not possess a suitable degree of firmness, it will resist such motions. Thus it is with soups and other liquid aliments, where Nature removes the watery part prior to the progress of digestion. Hence oils are digested with so much difficulty; and it is probable that jellies, and other glutinous matters, although containing the elements of nourishment in the highest state of concentration, are not digested without considerable labour; *first*, from their evading the grappling powers of the stomach, and, *secondly*, from their tenacity opposing the absorption of their fluid parts. For these reasons, it is maintained that the addition of isinglass, and other glutinous substances, to animal broths, with a view to render them more nutritive to invalids, is a pernicious practice. The texture of animal food is greatly influenced by the age, sex, habits, condition, diet, and manner of death of the animal. In proportion, generally, to the age, the flesh is coarser and more firm in texture, as almost every individual must have noticed in the eating of birds. If the flesh of mutton and lamb, beef and veal, be compared, they will be found of a different texture; the two young meats are of a more stringy, indivisible nature than the others, which makes them harder of digestion. Young animals differ, also, from old ones in the distribution of the fat, which, in the latter, is chiefly collected in masses or layers, external to the muscles; while, in the former, it is more interspersed among the muscular fibres, imparting to the flesh a marbled appearance, which is always desirable. The texture of food will likewise vary according to the wild or domesticated state of the animal, since the former is more dense, although very nutritious. Moreover, the sex modifies the quality, that of the female being invariably more delicate and finer grained than that of the entire male, whose fibres are denser. The flesh of hunted animals is peculiarly tender, and the same arises from any lingering death. The administration of vinegar prior to the killing of them has a similar effect. Incipient putrefaction strikingly ameliorates the rigidity of the animal fibre. Yet the circumstances enumerated as influencing the texture of food, and its degree of digestibility, are unimportant, when compared with the modifying powers of cookery. By this process, alimentary substances undergo a two-fold change;—their principles, are *chemically* modified, and their textures *mechanically* changed. Much, however, will depend upon the manner in which heat has been employed, and this will be generally explicable, by recollecting the kind of fuel most accessible to different countries.

1. *Boiling*. Here, the principles not properly soluble are rendered softer, more pulpy, and, consequently, easier of digestion. But the meat is deprived of some of its nutritive properties by the removal of a portion of its soluble constituents; the albumen and gelatin are also acted upon; the former being solidified, and the latter converted into a gelatinous substance. If, therefore, meat be boiled too long or too fast, there will be, where the albumen predominates, as in beef, a hard and indigestible mass, like an overboiled egg; or, where the gelatin exceeds, as in young meats, such as veal, a gelatinous substance alike injurious to the digestive organs. Hence young and viscid food, as veal, chickens, &c. is more wholesome roasted than boiled, and easier digested. Dr. Prout has justly observed, that the boiling temperature is too high for a great many of the processes of cooking, and that a lower temperature and a longer time, or a *species of infusion*, are better adapted for most of them. Thus it is that "*beef tea*" and "*mutton tea*"* are much more calculated for invalids than the broths of these meats. The loss occasioned by boiling partly depends upon the melting of the fat, but chiefly from the solution of the gelatine and osmazone. Mutton generally loses about one fifth, and beef

* Nothing can more strongly evince how much the English language demands strict revision and consequent improvement than the introduction of these barbarous phrases into the present work. Tea, in common conversation, implies an infusion of that Chinese plant in hot water:—how, then, can the infusion of beef, mutton, and sage, in a similar menstruum, be denominated tea, without a ludicrous absurdity? What should we think of a man who spoke of mutton-partridge, simply because his mutton was cooked as partridges are?—He would be thought mad, something like my Lord Peter, in the Tale of a Tub, who swore that a loaf of bread was both mutton and wine! Yet is this a case exactly analogous. If the ridiculous terms, here reprobated, sprang from the poverty of the language some excuse might be admitted, but it is not so; for beef, mutton, or sage-infusion would be perfectly correct, and unobjectionably short. It is now 115 years since Swift published a letter to Harley, Earl of Oxford, then Lord Treasurer, concerning the imperfect state of our language, and complaining that, in many instances, it offended against precision, sense, and grammar. What would the Dean have said, had he been told that, in the year 1826, a doctor of the University of Cambridge, and certainly one who has done her no discredit, would use, in a scientific work, such phrases as *beef, mutton, and sage-tea*.—*Rev.*

one fourth of its original weight. Boiling is particularly applicable to vegetables, rendering them more soluble in the stomach, and depriving them of a considerable quantity of *air*, which is so injurious to weak stomachs. But, even in this case, the operation may be carried to a hurtful extent; for instance, potatoes are frequently boiled to a dry and insipid powder instead of being preserved in a soft, gelatinous state, which retains their shape. Again, the cabbage tribe, and carrots, are commonly not boiled long enough, and in this condition they are highly indigestible. The quality of the water employed must also be attended to; for mutton, boiled in hard water is more tender and juicy than when soft is used; while vegetables are made harder, and less digestible.

2. *Roasting*. By this process the fibrine is corrugated, the albumen coagulated, the fat liquified, and the water evaporated. The surface first becomes brown, then scorched; and the tendinous parts are rendered softer and gluey. The meat should not be *over-done*, nor *under-dressed;* for though it may contain more nutriment, it will be less digestible from the density of its texture. Animal matter loses more by roasting than boiling. It has been stated, that by this latter process mutton loses one-fifth, and beef one fourth; but by the former these meats lose about one-third of their weight. In roasting, the loss arises from the melting of the fat, and the evaporation of the water; while the nutritious matter remains condensed in the cooked, solid; whereas, in boiling, the gelatine is partly abstracted. Roasted are, therefore, more nutritious than boiled meats; because it has been computed that, from the dissipation of the nutritive juices by boiling, one pound of *roasted* contains as much nourishment as two of *boiled* meat.

3. *Frying*. This is, perhaps, the most objectionable of all the culinary operations, since the heat is applied through the medium of boiling oil, or fat, which is rendered empyreumatic, and consequently extremely liable to disagree with the stomach.

4. *Broiling*. Here the sudden browning or hardening of the surface prevents the evaporation of the juices of the meat, and imparts to it a peculiar tenderness. It is the form selected by those that strive to invigorate themselves by *training*.

5. *Baking*. The peculiarity of this process depends upon the substance being heated in a confined space, which does not permit the escape of the fumes arising from it. The meat is, therefore, from the retention of its juices, made more sapid and tender; but baked meats are not so easily digested, on account of the greater retention of their oils, which are, moreover, in an empyreumatic state. Such dishes accordingly require the stimulus of various condiments to increase the digestive powers of the stomach.

*Of Condiments.* These are substances which, in themselves, are incapable of nourishing, but which, in concert with food, promote its digestion, or correct some of its deleterious qualities. The existence and necessity of such agents are far more universal and important than has been generally supposed.* The bitter principle which exists in grasses and other plants appears to be equally essential to the digestion of herbivorous animals. It acts as a natural stimulant; for a variety of experiments has shewn that it passes through the body without suffering either diminution, or mutation. No cattle will thrive upon grasses which are destitute of a portion of this vegetable principle; this fact has been most satisfactorily proved by Mr. Sinclair, in that magnificent work, the "*Hortus Gramineus Woburnensis.*" The value of slight bitters, and of condiments has been felt and acknowledged in all ages. From the different nature of condiment, it has been usually divided into three classes; viz. the *saline*, the *spicy* or *aromatic*, and the *oily*.

1. *Salt* appears to be a necessary and universal stimulus to animated beings; and its effects upon the vegetable and animal kingdoms have furnished the most interesting inquiries to the physiologist, chemist, physician, and agriculturist. It appears to be a natural stimulant to the digestive organs of all warm-blooded animals, and they are instinctively led to immense distances in the pursuit of it. To point out the advantages of salt as an article in diet, or to dwell upon the miseries that have sprung from the want of it, would be, indeed, a superfluous labour, since they are too notorious to be either questioned, or denied.

2. *Vinegar.* This acid, in small quantities, is a grateful and wholesome stimulant. It will often check the chemical fermentation of certain substances within the stomach, and prevent vegetable matter in its raw state from inducing flatulence, yet the use of it demands caution, and it is obviously noxious in some morbid states of the system. Fatty and gelatinous substances frequently appear to be rendered more digestible in the stomach by the addition of vinegar, although it is difficult to offer either a chemical or physiological explanation of the fact. The native vegetable acids may also be occasionally substituted. The addition of lemon juice to rich and glutinous soups makes

---

* See the Author's Pharmacologia, Ed. VI. Vol. I. p. 145.

them less liable to disagree with the stomach; and the custom of eating apple-sauce with pork, or goose, is attributable for its origin to the same cause.

3. *Aromatic Condiments* comprise the foreign spices, as pepper, cayenne pepper, cinnamon, nutmeg, cloves, ginger; and the indigenous herbs and roots, such as parsley, thyme, sage, garlic, leek, onion, horse-radish, mustard, &c. The former were not intended by nature for the natives of temperate climes, since they are heating, and highly stimulating. The dyspeptic invalid should be cautious in their use, because permanent mischief may result from temporary benefit. But mischievous as the abuse of aromatic condiments may be, it is insignificant in comparison with the custom of swallowing a quantity of brandy to obviate the upbraidings of a disordered stomach, or an increased libation of wine to counteract the distress which supervenes on a too copious meal:—as if drunkenness were an antidote for gluttony!

4. *Oil.* This, with butter, constitutes what is called the oleaginous condiments. Melted butter is, perhaps, the most injurious of all the inventions of cookery. Oil, when used in extremely small quantities, as a seasoning to salads, seems to prevent their running into fermentation, and, consequently, obviates flatulency.

*Of Drinks.* Liquid matter is as essentially necessary to the human frame as solid aliment; and the necessity of this supply, as well as its quantity, is indicated by a certain feeling denominated *thirst.* How much of diluents each person may require will depend upon individual peculiarity, climate, nature of the solid aliment, and likewise the customs of artificial life, which are too often at variance with common sense. Although fluids of the usual temperature are grateful and congenial to a healthy stomach, yet dyspeptic patients frequently need them to be raised to the temperature of the body; for the stomach, not having sufficient vital energy to establish re-action, falls into a state of collapse. But fluids, heated much above the temperature of the body, are equally injurious. Iced ones should not be taken, under any circumstances, by those whose stomachs are delicate, and especially after a meal, the digestion of which is thus retarded, or wholly prevented. Different aliments will require different quantities of liquid to assist their chymification. Animal food demands more drink than vegetable; roasted than boiled meat; and baked still more than roasted. Which is the most suitable period for taking liquids? By drinking *before* a meal, we place the stomach in a very unfit condition to perform its duties:—by drinking *during* a meal, we shall assist digestion, if the solid matter be of a nature that requires it; and we shall impede it, if the quantity taken render the mass too liquid. The best general rule, regardless of all preconceived theory, is that which is created by the sensations of the individual. Experience teaches, as Dr. W. Philip has stated, that "eating too fast causes thirst; for, the food being swallowed without a due admixture of saliva, the mass formed in the stomach is too dry." As hunger and thirst are, to a certain extent, incompatible sensations, it is probable that Nature intended the appetite for food to be first satisfied; and if the food possess that degree of succulence which characterizes digestible aliments, there will be no necessity for liquids. But, at all events, the quantity should be small. It is during the intervals of solid meals that the necessary drink ought to be taken; and both theory and experience demonstrate the advantage which attends a liquid repast about four or five hours after the solid repast. About this period the chyle has entered its proper vessels, and is flowing into the blood to undergo its final changes. Then it is that the stomach, having disposed of its charge, receives the wholesome draught with the greatest advantage; then it is that the blood, impregnated with new materials, requires the assistance of a diluent to complete their sanguification, and to carry off the superfluous matter; and it is then that the kidneys and the skin will need the aid of additional water to assist the performance of their functions. The common beverage of tea, or something analogous, doubtless originally suggested by an instinctive desire for liquids at this period, is thus sanctioned by theory, and its benefits established by experience.*

"*Water* is unquestionably the natural beverage of man; but any objection against the use of other beverages, founded on their artificial origin, I should at once repel by the same argument which has been adduced in defence of cookery. We are to consider man as he is, not as he might have been, had he never forsaken the rude path of Nature. I am willing to confess, that 'the more simply life is supported, and

* We believe that much of the *thirst* which is made the excuse for drink, both at and after dinner, arises either from a *bad habit*, established by long usage, or from eating too much in quantity, or food of an improper quality. We know several instances of dyspeptics who never could dine without drink both at and after eating; but who, on being put on a proper plan of diet, both as to kind and amount, required no drink at all, or not more than a cup of coffee when the repast was finished. We repeat it, then, that thirst, or the necessity for more than a very little drink with our principal meal, is, in general, a symptom that there is "something rotten in the constitution of Denmark."—*Editors*.

the less stimulus we use, the better; and that he is happy who considers water the best drink, and salt the best sauce:' But how rarely does a physician find a patient who has regulated his life by such a maxim! He is generally called upon to reform stomachs, already vitiated by bad habits, and which cannot, without much discipline, be reconciled to simple and healthy aliment. Under such circumstances, nothing can be more injudicious than abruptly to withdraw the accustomed stimuli, unless it can be shewn that they are absolutely injurious; a question which it will be my duty to investigate hereafter.

"The qualities of water differ essentially, according to the source from which it has been obtained; and those accustomed to this beverage are sensible to differences which wholly escape the observation of less experienced judges. How far the existence of foreign matter injures its salubrity, has been a subject of much controversy: the truth, perhaps, lies between the extremes; those who insist upon the necessity of distillation for its purification, and those who consider every description of water as alike salubrious, are, in my opinion, equally remote from truth. That the presence of minute quantities of earthy matter can become a source of disease, appears absurd; while it would be highly dangerous to deny the morbid tendency of water that holds putrescent animal or vegetable matter in solution, or which abounds in mineral impregnation.

"The usual varieties of common water were classed and defined by Celsus, and modern chemists have not found any reason to reject the arrangement—'*Aqua levissima pluvialis est; dein fontana, tum ex flumine, tum ex puteo; posthæc ex nive aut glacie, gravoir his ex lacu; gravissima ex palude.*'"—120.

Now the author enters into a consideration of the different kinds of water, but this we shall overlook for the sake of more interesting matter.

The juices and infusions of vegetable and animal substances constitute the second division of drinks.

1. *Toast Water.* By impregnating water with the soluble parts of toasted bread, it will frequently agree better with stomachs which are averse from the pure fluid, and it becomes slightly nutritive from retaining a certain portion of gum and starch. The water ought to be boiling, and drunk when sufficiently cool; since, by keeping, it acquires an unpleasant flavour.

2. *Barley Water.* This is an ancient, and much recommended drink. Barley affords less viscid potations than other grains. The invention of *pearl barley*, prepared by the removal of the husk, and afterwards rounded and polished in a mill, has greatly improved this grain. The granules consist chiefly of fecula, with portions of mucilage, gluten, and sugar, which water extracts by decoction; but the solution soon passes into the acetous fermentation. Lemon juice and sugar are grateful additions.

3. *Gruel.* Oats, without their cuticle, are called *groats.* In which state, or when ground into meal, they yield to water, by coction, the fecula they contain, and form a nutritious gruel, which is slightly aperient. Gruel becomes acescent after forty-eight hours, and it may be made of different degrees of consistence, according to the object in view. If as a demulcent drink, it should be thin; and Dr. Kitchener informs us it may be made by mixing well together, by degrees and in a pint basin, *one* table-spoonful of oatmeal with *three* of cold water, and then adding carefully a pint of boiling water: the whole is to be boiled for five minutes, stirring it constantly; and, finally, the liquor is to be strained through a hair-sieve. If something more substantial be demanded, the quantity of oatmeal may be doubled; or broth or milk may be substituted for water. Some mix butter with gruel, which is objectionable, where acidity of the stomach either prevails, or is suspected.

4. *Sage Tea.** The virtues of sage have been extolled, and depreciated; but, in the form of infusion, it certainly does possess some power in allaying the irritability of the stomach; and it is frequently exhibited by the Chinese as a tonic for debility of this organ. Sage, as well as balm tea, will furnish a salutary beverage on many occasions.

5. *Tea.* Two kinds of tea are imported into England, *black* and *green.* Each contains astringent and narcotic principles, but in very different proportions; the latter producing by far the most powerful influence upon the nervous system. Every narcotic being stimulating, tea exhilarates and refreshes; yet some, from certain peculiarities, experience, even from a single cup, nervous sensations, unnatural vigilance, and stomachic disorder, with feelings of depression. That the use of tea is generally beneficial is placed beyond doubt; but it ought to be taken about four hours after the principal meal, when, it will assist the ulterior stages of digestion, promote the insensible perspiration, and impart to the stomach a grateful stimulus. The addition of milk certainly diminishes the astringency of tea; while sugar may gratify the palate, but cannot modify the virtues of this infusion.

6. *Coffee.* Coffee, like tea, has had its enemies, and, probably, with equal injustice. It is more stimulant, and certainly exerts a different action in the nervous system, although the

---

* For the sake of convenience we use the author's phraseology in this instance.—*Rev.*

nature of this difference is not very obvious. After a meal, coffee does not create that disturbance in its digestion which occasionally arises after the drinking of tea; for it accelerates the operations of the stomach, and will frequently enable the dyspeptic to digest substances, such as fat and oily aliments, which would otherwise be the cause of much disorder. Coffee, taken immediately after dinner, as practised by the French, doubtless counteracts the probable evil effects of their diet. This infusion, as well as tea, has certainly an antisoporific effect on many individuals; and imparts an activity to the mind which is incompatible with sleep. It is generally admitted to possess the power of obviating the effects of narcotics; and hence it is so used by the Turks. Where coffee is intended to promote digestion, it should be carefully made by infusion, because decoction dissipates its aroma. Milk may well be omitted, while sugar, or rather sugar-candy, is allowable. There are some persons in whom coffee induces acidity in the stomach.

7. *Chocolate.* This liquid contains a large quantity of nutritive matter, and should be regarded as food rather than drink. It is prepared by reducing the cocoa nut into paste, with sugar, milk, or eggs: it is also frequently mixed with different aromatics, the most common of which is the *vanilla*, a substance very liable to disagree with the stomach, and to produce a train of nervous symptoms. As a common beverage, chocolate is highly objectionable; it contains an oil which is difficult of assimilation; it likewise oppresses the stomach; and this effect is increased by too much heat in the preparation.

8. *Cocoa* is usually considered as a substitute for chocolate, and, containing less nutritious matter, it is not so objectionable; for the oily matter exists only in small quantities, and, consequently, is not so likely to disagree with the stomach.

9. *Whey* is a delightful beverage; but its nature and operation cannot be well understood prior to the investigation of the composition of milk.

There are certain saline solutions which are frequently employed as drinks, and deserve some attention; such are *imperial* and *soda water*.

1. *Imperial.* This is a solution of cream of tartar, flavoured with lemon peel. It ought never to be used, except as a medicine; for, if taken as an ordinary drink, it is apt to retard digestion. As an article of diet, it is only admissible under circumstances of robust health, and where a large quantity of animal food has been eaten.

2. *Soda Water*. The drinking of this during, or immediately after, dinner, is a pregnant source of dyspepsia. By inflating the stomach at such a period, we inevitably counteract those muscular contractions of its coats which are essential to chymification. The quantity of soda thus introduced scarcely deserves notice: with the exception of the *carbonic acid gas*, it may be regarded as water, more mischievous, only, in consequence of the exhilarating quality inducing the individual to swallow it at a time when he should not require the more simple fluid. Late discoveries have shewn, that the *carbonic acid* exists in a liquid state in soda water; when, therefore, it is hastily drunk, it robs the stomach of a certain portion of heat as it passes from a liquid into a gaseous state; and, consequently, cools, as well as distends that organ.

Having proceeded thus far, Dr. Paris next examines into the nature and qualities of fermented liquors, and for this subject, most ably handled, we must unavoidably refer to the work itself, and we do so the more confidently, because we have, in a recent Number* of our Journal, devoted an article to ancient and modern wines.

"Malt liquors differ from wines in several essential points; they contain a much larger proportion of nutritive matter, and a less proportion of spirit; while they contain a peculiar bitter and narcotic principle derived from the hop. It would appear that the extractive matter furnished by the malt is highly nutritive; and we accordingly find, that those persons addicted to such potations are, in general, fat.† Where, however, they are indulged in to any extent, without a corresponding degree of exercise, they induce a plethoric state of the body, and all the diseases consequent upon such a condition." "Ale, when taken without precaution, is liable to disturb the digestive organs. The addition of the hop increases the value of the liquor, by the grateful stimulus which it imparts, and in some measure redeems it from those vices with which it might otherwise be charged. To those, therefore, whose diet is not very nutritive, ale may be considered, not only as an innocent, but as a salubrious article; and happy is that country whose labouring classes prefer such a beverage to the mischievous potations of ardent spirit. These remarks, however, cannot apply to those classes of the community who 'fare sumptuously every day.' They will not require

* Henderson on Ancient and Modern Wines, No. 8, 1826, April. New Series.

"† This fact is so generally admitted by all those who are skilled in the art of *training*, that a quantity of ale is taken at every meal by the pugilist who is endeavouring *to screw himself up to his fullest strength*. Jackson, the celebrated trainer, affirms, if any person accustomed to drink wine would but try malt liquor for a month, he would find himself so much the better for it, that he would soon take the one, and abandon the other."

a nutritive potation of such a character; and light wines have accordingly, in these days of luxury, very properly superseded its use: but I am not disposed to extend this remark to its more humble companion, '*table beer.*' I regard its dismissal from the tables of the great as a matter of regret; its slight, but invigorating, bitter is much better adapted to promote digestion than its more costly substitutes. But it should be soft and mild; for, when stale and hard, it is likely to disturb the bowels, and occasion effects the very opposite to those it is intended to produce. Nor ought it to have too great a proportion of hops, but should be thoroughly fermented and purified. Sydenham always took a glass of small beer at his meals, and he considered it as a preservative against gravel.

"The great division of malt liquors is into small beer, ale, and porter." 147—8.

We are much pleased with the above observations, as well as with those which follow on ale, porter, and ardent spirits; and to the truth of them we have long been converts.

It is now that Dr. Paris attempts an estimate of the nutritive and digestible qualities of several species of aliment, as derived from the application of the physiological and chemical principles established in his preceding pages. Compelled as we are, by defined limits, to disregard his remarks on milk, which, although fluid, is, in fact, a mixture of solid and liquid aliment, and separable into three proximate ingredients, *cream*, *curd*, and *whey*, we proceed in regular analytic order.

*Eggs*, in point of nutriment and digestibility, may be classed next to milk; but their qualities will greatly depend upon the manner in which they are cooked. When raw, they are certainly not so easily digested as when lightly boiled, so as slightly to coagulate their albuminous principle; but if this be carried too far, they are converted into a hard mass, which requires all the powers of the stomach for its chymification. This is, however much accelerated by the use of vinegar as a condiment. Eggs singularly disorder some stomachs, even in the smallest quantity; while, in others, they are harmless:—When raw, they are laxative; and, when cooked, apt to engender constipation.

*Fish* has been generally considered as holding a middle rank between the flesh of warm-blooded animals and vegetable food. It is less nutritive than mutton or beef; but the health and vigour of the inhabitants of fishing-towns, demonstrate that it is sufficiently nourishing for active life. To satisfy the appetite, a large quantity is requisite; and the appetite returns sooner than after a diet of meat. Nor does fish produce the same stimulus to the body; the pulse is not strengthened as from a repast of flesh; and that febrile excitement is wanting which attends the digestion of the more nutritious viands. Hence, fish affords a most valuable article of diet to invalids, labouring under particular disorders; since it furnishes a chyle moderately nutritive, yet not highly stimulant. Fish does not need a laborious operation of the stomach; and is also free from the objections urged against liquid or gelatinous food. Such a diet is not calculated to restore debilitated habits, and, in such cases, is altogether inadmissible, except when the digestive powers are unable to convert stronger aliment into chyle. From its low stimulant power, fish requires condiments; and, consequently, salt appears to be an essential accompaniment.

"Fish have been arranged under three divisions; viz. *fresh-water fish, salt-water fish, and shell fish.* But, since the value of these animals, as articles of food, has an intimate relation to the colour and texture of their muscles, and to their gelatinous or oily qualities, it will be expedient to consider their several varieties, with reference to such conditions. Turbot, cod, whiting, haddock, flounder, and sole, are the least heating of the more nutritive species; and the flakiness of the fish, and its opaque appearance after being cooked, may be considered as true indications of its goodness; for when the muscles remain semi-transparent and bluish, after sufficient boiling, we may reject it as inferior in value, or not in season. When the fish is in high perfection, there is also a layer of white, curdy matter, resembling coagulated albumen, interposed between its flakes. The whiting is well adapted for weak stomachs, on account of the little viscidity which it possesses; it is, at the same time, tender, white, and delicate, and conveys sufficient nutriment, with but little stimulus, to the system. The haddock much resembles it, but is firmer in texture. Cod has a more dense fibre than the two former, and contains, also, more glutinous matter; it is an excellent aliment, but, upon the whole, is not quite so digestible as whiting or haddock. It is generally preferred when large; but such fish are frequently coarse. The haddock is certainly better when it does not exceed a middling size." 159.

For much more valuable information on this interesting subject, the reader is referred to the book itself, and we venture to say he will not be disappointed.*

---

* With every deference and respect for the opinions, both of Dr. Paris, and the talented, as well as experienced writer of this article, the Editor of this Journal differs from the sentiments of both, in respect to fish as an article of food in dyspepsia. He appeals to the experience of dyspeptics themselves (and there are many of them in the profession) whether fish, even the plainest white fish, is not much more apt to disagree with the stomach than animal food. Some of the severest attacks of indigestion which he ever witnessed were caused by fish. With butter, it is injurious—without butter, it is insipid. It is allowed that fish requires condiments; but condiments

*Birds.* There is a great variety in the qualities of the food of this class of animals, with respect to nourishment, stimulus, and digestibility. The whiter meat of domesticated birds, as the wings and breasts of chickens, contains less nutriment, and is less digestible, than that which is furnished by wild birds, as the partridge &c.; yet, the former is less heating and stimulating. These circumstances must direct the opinions of the medical practitioner; and the same observations will apply to the flesh of quadrupeds; for that which is dark-coloured, and possesses a large proportion of fibrin, as venison, &c. is easily disposed of by the stomach, and produces a large quantity of highly stimulating chyle. The whiter meats, on the contrary, are detained longer there, and generate a less stimulant chyle. The former, therefore, will be more readily digested by weak persons, while the latter will frequently run into acetous fermentation; but they may, nevertheless, be preferable on many occasions, because they impart less stimulus to the general system.

The next sixteen pages of this work are appropriated to the consideration of "*Farinaceous Aliments;*" such as wheaten bread, and adulterations of it; leaven; barm; panification; kinds of bread; barley, rye, and oaten bread; puddings, pancakes, &c.; potatoe, rice; leguminous vegetables, or pulses; and then follow observations on peas, nuts, esculent roots, and herbs. For all these we must refer to the book itself, those who are unacquainted with such matters.

*Fruits.* These are generally regarded as luxuries; and, from their abuse, they may be classed rather under poisons than aliments. Nothing can be more injurious than an excess of them in the form of dessert, or otherwise; but, judiciously taken, they contribute to health, and appear to be, at proper seasons, a desirably cooling and antiseptic food. Fruits may be arranged under the following heads: stone fruits, the apple species, small-seeded fruits, small berries, and farinaceous fruits. The stone fruits are the least digestible, but much depends upon the unripe state in which they are eaten; yet still they are very liable to undergo fermentation in the stomach. The hard pulp of certain plums remains also in the alimentary canal for a long time, and is frequently ejected without material change. The ripe peach is the most delicious, and one of the most digestible of the stone fruits. The apricot is equally wholesome, while the nectarine is apt to disagree with particular stomachs.

---

themselves are very generally prejudicial in dyspepsia. Upon the whole, he warns the dyspeptic invalid against fish—and he appeals to their own feelings after eating it, for the justness of the objection.

Cherries are far less digestible: their pulpy texture and skins are not easily disposed of by the stomach; and, as the sweetest contain a considerable excess of acid, they may be objectionable in some, and desirable in other cases. The apple species is not so dilute and watery, and is consequently less likely to pass into noxious fermentation; but its texture is firmer, and being, from this circumstance, longer retained in the stomach, it often proves indigestible. The same observations apply in a minor degree to pears. The orange, when perfectly ripe, may be allowed to the fastidious dyspeptic; yet the white, or inner skin, should be scrupulously rejected, for it is not more digestible than leather. The small-seeded fruits are undoubtedly the most wholesome; and of those, the ripe strawberry and raspberry deserve the first rank. The grape is likewise cooling and antiseptic, but the husks and seeds should be discarded. The gooseberry is less wholesome, especially from the indigestibility of the skin, which is too frequently swallowed. The fruits to be classed under the head of small berries, are the cranberry, the bilberry, and the red whortleberry. These are seldom eaten, except when baked, and in that state their acescency seldom proves injurious. The farinaceous fruits are universally hurtful. The melon, which is the principal one, is very apt to disagree with weak stomachs, and should never be eaten after dinner, without a plentiful supply of salt and pepper. Mornings and evenings are the most proper periods for indulgence in fruit. On some occasions, it may be taken with advantage at breakfast, or three hours before dinner, and it affords a light and agreeable repast if enjoyed an hour before bed-time. But these regulations are to be influenced by circumstances which no general rule can possibly embrace. By cookery, fruit, otherwise unwholesome, may be converted into a safe and useful aliment. Apples, when baked, afford a pleasant entertainment; and from their laxative qualities are well adapted to certain cases of dyspepsia. Fruit pies, if the pastry be entirely rejected, are valuable articles of diet. Dried fruits are by no means so useful or safe as is generally imagined; the quantity of sugar which enters into their composition disposes them to fermentation.

A few words remain to be expressed upon the subject of the intermixture of various aliments; or, in other language, the too prevailing fashion of introducing at meals an almost indefinite succession of incompatible dishes. The stomach being distended with soup, the digestion of which, from the very nature of the operations which are necessary for its completion, would in itself be a sufficient labour for that organ, is next tempted with fish, rendered indigestible by its sauces; then with flesh and fowl;—finally "the vegetable world is ransacked from the *cryptogamia* upwards;" and to this miscellaneous aggregate are added the pernicious pasticcios of the pastry-cook, and the complex combinations of the confectioner. It is not to one or two good dishes, even abundantly indulged in, but to the overloading of the stomach, that such strong objections are to be urged. Nine persons in ten swallow as much soup and fish as would amply suffice for a meal, and as far as these are concerned, would rise not only satisfied but saturated; yet a new stimulus appears in the form of stewed beef, or côtelettes à la suprême: then comes a Bayonne or Westphalia ham, or a pickled tongue, or some analogous salted, but proportionately indigestible dish, and of each enough is eaten for a single meal. Alas! this is not all; game follows; and to this again succeed the sweets, and a quantity of cheese; and the whole is crowned with a variety of flatulent fruits and indigestible knick-knacks, included under the name of dessert, and accompanied with a mountain of sponge cake. Thus the stomach receives not one full meal, but a succession of meals, rapidly following each other.

"But it may be said, that this is a mere tirade against quantity; against over-distention of the stomach; that it argues nothing against variety of food, provided the sum of all the dishes does not exceed that which might be taken of any single one. Without availing myself of the argument so usually applied against plurality of food, that 'it induces us to eat too much,' I will meet the question upon fair grounds. It is evident that the different varieties of food require very different exertions of the stomach for their digestion; it may be that the gastric juice varies in composition, according to the specific nature of the stimulus which excites the vessels to secrete it; but of this we are uncertain, nor is it essential to the argument: it is sufficient to know, that one species of food is passed into the duodenum in a chymified state in half the time which is required to effect the same change in another. Where, then, the stomach is charged with contents which do not harmonize with each other in this respect, we shall have the several parts of the mixed mass at the same time in different stages of digestion: one part will, therefore, be retained beyond the period destined for its expulsion, while another will be hurried forward before its change has been sufficiently completed. It is then highly expedient, particularly for those with weak stomachs, to eat but one species of food, so that it may be all digested and expelled at nearly the same period of time; that when the duodenal digestion has been fully established, the operations of the stomach shall have ceased. I have, on a former occasion, insisted upon the importance of such a regulation, and it now leads one to make some remarks upon the periods best adapted for our meals, and upon the intervals which should be allowed between them."—185.

*On the Periods best adapted for Meals, and on the Intervals which should elapse between each.* An invalid may safely take three frugal meals; or, on some occasions, even four, provided a certain quantity of exercise be enjoyed.

1. *Breakfast.* This is, perhaps, the most natural, and not the least important meal; since, from the long period of abstinence, the stomach ought to be in a condition to receive aliment. But this is not always the fact; the gastric juice does not appear to be secreted in any quantity during sleep, while the muscular energies of the stomach, although invigorated by repose, are not immediately called into action. It is, therefore, advisable, and the practice is confirmed by experience, to allow an interval to pass prior to breakfasting. The solidity of the breakfast should be regulated by the labour and exercise to be undergone, and the time of dining. Where the dinner hour is late, a more nutritious meal may be recommended, to supersede the necessity of *luncheon,* or what the French designate *un dejeuner à la fourchette.* Yet it must not be forgotten that dyspeptic invalids are frequently incommoded by such a repast, if it be copious. Heartburn is a common effect, especially if the heavy breakfast be accompanied with much diluting liquid. Hence a question has arisen as to the propriety of taking much fluid on such occasions. It will not be difficult to show the reasons why liquids are essentially necessary at breakfast; because, overlooking obvious ones, there is a certain acrimony and rankness in all the secretions at that period; and, likewise, the breath has commonly a peculiar taint in the morning, which is not observable at subsequent times of the day. Every physician must have been consulted upon the propriety of taking meat, tea, or coffee, at breakfast. A person who has not strong powers of digestion, is frequently distressed by the common association of tea with bread and butter, or, what is more injurious, with hot buttered toast or muffin; the oily part of which is separated by the heat of the liquid, remains in the stomach, and produces, in its cardiac orifice, an irritation tantamount to heartburn. Here dry toast is preferable. New bread, or spongy rolls, should be carefully avoided. Tea, to many individuals, is a beverage which contains too little nutriment; and when so, barley water, or a thin gruel, is a very useful substitute. A constant acidity between breakfast and dinner, but at no other period, has been removed by eating toasted bread, with a slice of the lean part of cold mutton, and a large cup of warm barley-water for the purpose of dilution. Hard eggs, although they require a long time for their digestion, are not generally offensive to the stomach; and consequently may be taken with propriety at breakfast whenever the hour of dinner is late.

2. *Dinner*. This is the principal meal of modern times, and at which every species of luxurious gratification is practised. Invalids should dine in the middle of the day, or at two or three o'clock. The exact period, however, must be directed by the physician with reference to the necessary habits of his patient, the nature and time of his breakfast; and, above all, to the rapidity or tardiness of his digestion. For instance, a gentleman dining at three, had scarcely any appetite, and constantly experienced a sensation of weight and uneasiness afterwards; but was cured by always taking his dinner at six o'clock, because, when he dined at the former hour, his breakfast had not been duly digested in consequence of his digestion being remarkably slow.

3. *Tea*. This subject has already been considered; and, where it is practicable, exercise should follow the repast.

4. *Supper*. When Queen Elizabeth flourished, the nobility and gentry were accustomed to dine at eleven, to sup between five and six, and to go to bed at ten. Any argument, therefore, in favour of this meal, founded upon the healthy condition of our ancestors, must be fallacious. By supper, in modern times, a late meal before bed-time is understood. But as sleep is not favourable to every stage of digestion, it is very questionable whether retiring to rest with a full stomach can, under any circumstances, be salutary. During chymification a person so situated may perhaps sleep quietly, unless indeed the morbid distention of the stomach should impede respiration, and occasion distress; but when the food has left the stomach, and chylification and sanguification have been established, the natural propensity of the body is for activity, and the invalid awakes at this period, and remains in a feverish state for some hours. Upon this general principle, then, suppers are to be avoided; that is, *hearty* ones, which require for their digestion the active powers of the stomach. The same cannot be urged against a light repast, which is generally useful to dyspeptics; and it has been truly and facetiously observed, that "some invalids need not put on their night-caps, if they do not first bribe their stomachs to good behaviour." An egg lightly boiled, or a piece of dry toast, with a small quantity of white wine negus, will often secure a tranquil night. Among the intellectual part of the community, there has ever existed a strong predilection in favour of suppers; for the labour of the day has been performed; the hour is sacred to conviviality, and is one not likely to be interrupted by business. To those in health, such indulgencies may occasionally be allowed; yet the physician should be cautious in giving his sanction to their wholesomeness.*

*On the Quantity of Food that ought to be taken at different Meals.* We cannot deprive our readers of the pleasure of perusing the following no less happy than just, observations. We have not shrunk, and we never will shrink, from the labour of analysis; but there are times when it is peculiarly proper to permit an author to appear in his own language, since not to concede this to him, is to rob him of the merit of his best passages. Our wish is to blazon forth, and not to darken intellectual light.

"Mr. Abernethy says, that 'it would be well if the public would follow the advice of Mr. Addison, given in the Spectator, of reading the writings of L. Cornaro; who, having naturally a weak constitution, which he seemed to have ruined by intemperance, so that he was expected to die at the age of thirty-five, did at that period adopt a strict regimen, allowing himself only *twelve ounces* of food daily.' When I see the habits of Cornaro so incessantly introduced as an example for imitation, and as the standard of dietetic perfection, I am really inclined to ask with Feyjoo—did God create Lewis Cornaro to be a rule for all mankind in what they were to eat and drink? Nothing can be more absurd than to establish a rule of weight and measure upon such occasions.

* In regard to suppers, the wisest method is to be guided by the feelings of the patient. Some dislike suppers, and take them with reluctance, while others, toward night, become hungry, and endure an aching, and a disagreeable emptiness of the stomach, which are commonly removed by eating a little animal food, and drinking a glass of ale, or of wine and water. We wonder the author has not interdicted the use of green tea, which is exceedingly pernicious to all dyspeptics, and indeed often produces cardialgia, and gastrodynia in the healthy. With respect to drinking during dinner, the best general rule is to avoid the practice, provided it can be done without distress, but certainly not otherwise, yet great moderation ought to be observed. If malt liquor be drunk after dinner, it should be very slowly, and not exceeding twelve ounces in half an hour, since, quickly taken, it unpleasantly distends and disorders the stomach. The same observation applies to wine;—for instance, a glass every quarter of an hour, not advancing beyond three, or four glasses of wine, and of which madeira and sherry are the most salutary. But where both malt liquor, and wine are indulged in, the proper plan is to drink the former first, and when an hour and a half have expired to commence with the latter. For, it is a well known fact, that, about two hours after dinner, the dyspeptic sufferer often experiences what he expressively terms, "*a terrible sinking of the stomach*," and which doubtless arises from the weakness consequent to chymification. Now wine taken prior to this period, and as we have advised, does, to our certain knowledge, either prevent, or overcome this much dreaded sinking.—*Rev.*

Individuals differ from each other so widely in their capacities for food, that to attempt the construction of a universal standard, is little less absurd than the practice of the philosophical tailors of Laputa, who wrought by mathematical calculation, and entertained a supreme contempt for those humble and illiterate fashioners who went to work by measuring the person of their customer: but Gulliver tells us, that the worst clothes he ever wore were constructed on abstract principles. How then, it may be asked, shall we be able to direct the proportion of food which it may be proper for an invalid to take? I shall answer this question in the words of Dr. Philip, whose opinion so exactly coincides with my own experience, that it would be difficult to discover a more appropriate manner of expressing it. 'The dyspeptic should carefully attend to the first feeling of satiety. There is a moment when the relish given by the appetite ceases: a single mouthful taken after this, oppresses a weak stomach. If he eats slowly, and carefully attends to this feeling, he will never overload the stomach.' But that such an indication may not deceive him, let him remember *to eat slowly*. This is an important condition; for when we eat too fast, we introduce a greater quantity of food into the stomach than the gastric juice can at once combine with; the consequence of which is, that hunger may continue for some time, after the stomach has received more than would be sufficient, under other circumstances, to induce satiety. The advantage of such a rule over every artificial method by weight and measure, must be obvious; for it will equally apply to every person, under whatever condition or circumstances he may be placed. If he be of sedentary habits, the feeling of satiety will be sooner induced; and if a concurrence of circumstances should have invigorated his digestive powers, he will find no difficulty in apportioning the increase of his food, so as to meet the exigencies of the occasion."—196.

Although we all take more solid food in health than may be necessary for supporting the body; yet too great an abstinence will also tend to weaken and distress both mind and body. The effects of low feeding are obvious in the diseases of the poor and ill-fed classes in many parts of England and Ireland; and these are still more striking in those districts where the food is chiefly or entirely vegetable, and, therefore, less nutritious. The obstinate fasting of maniacs often produces a disease resembling the sea scurvy. But those who, from their situation in life, constantly exceed the proper standard of diet, will preserve their health by occasionally abstaining from food, or rather, by reducing the usual quantity, and living low. This subject deserves farther consideration. A person after an attack of acute disease, when his appetite returns, is in the condition of a pugilist who is about to commence a system of *training;* but he is still more obnoxious to the evils presumable to accrue from excessive feeding. Weak, emaciated, without any disease, with a voracious appetite, he eats frequently and largely to recover his lost strength. For a time all is sunshine; his vigour increases daily; when, suddenly and unexpectedly, his appetite fails, he becomes again unwell, and fever, or some other mischief, assails him.

Any sudden transition from established habits, with regard to the quantity and quality of food, is injudicious. Individuals ought not to leap at once from the situation which causes pain, or creates alarm, but should quietly descend by safe and gradual steps. After long fasting also, great caution must be observed in the indulgence of aliments. When a famine occurred in the city of Bochara, those, who had lived on roots and herbs, died, because they filled themselves immoderately on their return to bread and meat. The advantages, derivable from rendering food grateful to invalids, are so striking, that the most digestible aliment, if it excite aversion, is more injurious than that which, though otherwise objectionable, gratifies the palate. If feelings of disgust or aversion are produced, the stomach will never act with healthy energy on the ingesta; while, in cases of extreme dislike, they are either rejected, or discharged almost unchanged. On the contrary, the gratification which attends a favourite meal is, in itself, a specific stimulus to the digestive organs, and especially in weak habits.

*Conduct to be pursued previous and subsequent to Meals.* Exercise in the open air is essential to the well-being of every person; but its degree must be regulated by individual circumstances. The interval between breakfast and dinner is the period for active exertion, while the enjoyment of it, when unattended with severe fatigue, will invigorate all the functions. This, too, is the period for the mind to direct its energies successfully, recollecting that *the valetudinarian or dyspeptic ought never to take his principal meal in a state of fatigue.* But the reverse of this salutary rule is practised by numbers, and by none more tenaciously than by the invalid merchant, the banker, the attorney, and the government clerk. Nay, dyspeptic patients have been restored to health by pursuing a different conduct. Remember that moderate exercise prior to dining is salutary; and that it is the abuse of it which is decried. Every one should inhale the open air anteriorly to a full meal, and undergo suitable exertion without consequent fatigue. Horse exercise is unquestionably beneficial, but it should not supersede that of walking. The occupation of *digging* is salutary; and especially to dyspeptics by agitating the abdominal organs. Constipation of the bowels is one of the great evils arising from sedentary habits; and this may, to a certain degree, be remedied, by standing for a definite period, instead of sitting, before a desk.

"If exercise be useful during the period of sanguification, pure air is no less so; and I shall take this opportunity of entering my protest against the introduction of *gas* into the interior of our houses. *Carburetted hydrogen* is a deadly poison; and even in a state of great dilution, it is capable of exerting a very baneful effect upon the general system. I have been consulted on several occasions for pains in the head, nausea, and distressing languor, which evidently had been produced by the persons inhaling the unburnt *gas* in the boxes of our theatres."

After relating the great sufferings and apparent danger of Sir Humphrey Davy, produced by the voluntary inspiration of *carburetted hydrogen gas*, the author concludes thus:—

"After this proof of the poisonous nature of carburetted hydrogen, —after the cases of sickness and headach which have occurred, in consequence of its inhalation at the theatre, am I not borne out in my opinion, that *its introduction into our apartments is fraught with danger?*" 206.

*Of the Influence of different Aliments in modifying the Appearances of the Discharges of the Body.* The external characters of the feculent discharges may be said to announce the healthy or diseased state of the digestive functions, with as much certainty as the pulse does that of the sanguiferous system. But, to deduce safe conclusions from such data, the nature and extent of the changes produced in these discharges by different aliments and medicines, must be well understood. The air has also the effect of altering the colour of the fæces, and hence they should be inspected without delay. This applies with great force to the stools of infants, which, perfectly yellow when voided, speedily assume a green appearance; and probably from the spontaneous decomposition of the bile.

Certain green vegetables, particularly spinach, impart a green hue to the fæces, that may be mistaken for vitiated bile; and beet-root will likewise give a colour. Pale-coloured evacuations, as if the bile were imperfectly secreted, may arise from drinking a considerable quantity of milk. Where the food has been of a very complicated description, the fæces will generally assume a crude and diversified character, owing, perhaps, to the several parts not having undergone the same degree of digestion. Where much stimulant drink has been used, and the person subjected to long fasting, or much labour, or has perspired profusely, the fæces acquire a hardened state. It is essential to know, that certain parts, both of animal and vegetable substances, pass through the alimentary canal without any change: *e.g.* the skin and seeds of fruits, &c. Cheese is also very apt to eject itself altogether undigested; and that formed into a ball by the action of the intestines, or a portion of caseous matter actually formed there from the drinking of milk and coagulated by the gastric juices, has been mistaken for an intestinal concretion. Concretions of oat-seeds are not unusually evacuated by the inhabitants of Scotland and Lancashire, where the oat-cake is in common use among the lower classes. The spawn of lobsters, a very indigestible substance, has also occasioned similar deceptious appearances. Magnesia, when repeatedly taken, will, by the assistance of a little animal mucus, become consolidated into masses of formidable magnitude. Certain medicines likewise change the colour of the excrements; iron imparts a black, and magnesia a white appearance. These circumstances* ought to be attended to, where it is an object to ascertain the state of the biliary secretion by the colour of the stools; and it would be judicious, on such occasions, to restrict patients to a diet that has no power to alter the appearances of the alvine discharges.

The intelligent author has now brought to a conclusion his history of Alimentary Substances. It will readily be perceived, he observes, that the terms *digestible* and *indigestible*, as applied to particular species of food, are but relative in their import, and depending upon the circumstances which he has endeavoured to investigate. The theory of digestion, and the history of Alimentary Substances, are, in his opinion, so intimately connected with the diseases to which our organs are

---

* The author has very properly directed attention to the colour of the alvine discharges, which is, at times, a great source of fallacy. Doubtless the colour of them is occasionally indicative of morbid conditions, yet the discolouration is often produced by various aliments, fluids, and medicines; and, if this fact be overlooked, incorrect opinions may be formed, and improper treatment recommended. Port wine and claret will make the stools of a black appearance in those unaccustomed to their use; and so will bottled London porter, unquestionably, from the intermixture of certain noxious ingredients. Again, the fine yellow colour of the evacuations, believed to arise from the exhibition of the blue pill, or calomel, can be generated by eating dried French plums, or prunes, as we have ascertained in numerous instances; while the apparent bilious tinge of a coated tongue may easily be caused by chewing a little gingerbread. Such are the trifles, recorded by Dr. Paris and ourselves, which sometimes unknown, and sometimes forgotten, mix themselves with the practice of physic, and create theories that are begotten on error, and founded upon misconception, and slope the way to the cures of diseases which never had any existence. This is, indeed, a fertile subject, and may, hereafter, be resumed.—*Rev.*

exposed, that, without a thorough knowledge of the former, we cannot expect to understand the phenomena of the latter, nor to establish a rational and successful system of treatment for the prevention and cure of Dyspepsia. He now proceeds to the third division of his work, which will embrace the history of Indigestion in all its forms and stages, and in which he hopes to turn the principles, already developed, to a practical advantage.

### PART III.—*Of Indigestion.*

*Dyspepsia* and *indigestion* are here considered as synonimous terms; and, notwithstanding the distinction of Dr. Philip, with obvious propriety, since the word *indigestion* is evidently a literal English translation of the Greek compound *dyspepsia.*

"I define indigestion to be *a primary disease, in which one or more of the several processes by which food is converted into blood, are imperfectly or improperly performed, in consequence either of functional aberrations, or organic lesion.** This definition may, perhaps, be opposed,

* Dr. Paris has created no little confusion here by attempting too much. If the definition had terminated with "*functional aberration*" all would have been well; but, by including "*organic lesion,*" it involves a contradiction, and comprises a singular error in pathological reasoning. What! is indigestion defined to be always a primary disease, and yet, sometimes, a consequence of organic lesion? To say indigestion is a consequence of functional aberration is intelligible enough, because such aberration constitutes the disease itself. In the first instance, indigestion is universally understood to be a functional, and consequently a primary disorder. True it does occasionally excite organic lesion of some part or other, although not so frequently as is usually represented, but in no manner will this admission assist the definition in discussion. For if indigestion should occasion organic mischief, that mischief will be the consequence, and not the cause of that malady; or, if the indigestion be generated by organic lesion, as the author affirms it sometimes is, why then it is not a primary, but a secondary disease. In fact, indigestion must be, in its early stages, invariably a functional disturbance; for, if it were the reverse, it could not, without absurdity, be called indigestion, since such an indigestion would be a mere symptom of an organic affection. Diseases are designated according to their actual nature, and not to their obvious indications. If a man suffer from simple irritability of the stomach, such is its right name, here it is certainly primary; while, if the irritability be an indisputable symptom of a diseased pylorus, such would be the distinctive appellation. So is it with indigestion, a primary and functional disorder, commonly ending favourably; yet, at times, giving rise to structural disease, which is, and ever must be, under the circumstances dwelt upon, a consequence of indigestion, and not indigestion a consequence of it, agreeably to this notable definition. But it may be alleged, the indigestion is apparent, while the organic lesion, however existing, may be thoroughly masked, and consequently unsuspected. Granted! yet this allegation will avail the author nothing. What produced on the ground of its too comprehensive signification; but I may observe, that however extensive may be the series of symptoms which are thus included under one general head, they will afford, when viewed collectively, sufficient evidence of their relation with the digestive process; although, on a loose and hasty observation, they may not present any general principle of dependency and connexion; if they appear disunited, let the practitioner suspect that he has never viewed them with sufficient reference to that physiological harmony which subsists between the organs in which they arise. Acidity of stomach and urinary depositions are equally indicative of deranged digestion; but the mind that is not acquainted with the relations of the stomach and kidneys, or with the connexion which subsists between the formation of perfect chyle and the discharge of natural urine, will not be disposed to arrange symptoms, so apparently remote in their reliance, under one common head." 214.

"From the universal sympathy which the stomach entertains for every part of the living body, its functions may become impeded or perverted from the existence of diseases which originate in organs with which it has no immediate connexion; an affection of the head, or even a disease in the urethra, may create sickness, loss of appetite, or a suspension in the digestive process; but such phenomena are not to be confounded with the primary symptoms of dyspepsia; they are affections of sympathy or induction, and will require very different treatment."— "I am strongly inclined to think that physicians of the present day are too apt to accuse the alimentary functions of offences which should be charged on other organs." 215.

1. *Imperfect Chymification.* The symptoms which arise from this cause are generally those which first indicate the approach of indigestion, and they frequently recur at intervals, for a considerable period, without occasioning any constitutional disturbance, or even a degree of local distress sufficient to awake alarm. In some cases, indeed, they are only produced by particular aliments, or under peculiar circumstances; but in others, they follow the ingestion of every species of food, although their violence is usually influenced by the quality and quantity of the meal. In this latter case, a morbid state of the stomach exists, which ought to be remedied without delay. We must ascertain whether a peculiar idiosyncrasy of the

---

the indigestion? What occasioned the organic lesion? Which had the priority? The indigestion? Then Dr. Paris is wrong, for it generated the organic lesion, whereas he maintains the direct contrary! Was it the organic lesion? Here Dr. Paris is equally wrong, since with what propriety can he define indigestion, and without any qualification whatsoever, to be a primary disease, when the exciting cause of it is organic lesion.—*Rev.*

stomach prevails in instances where indigestion is caused only by some particular aliment; or whether there be a debilitated condition of that organ, that incapacitates it from digesting any food demanding considerable powers for its chymification. The mucous membranes of certain stomachs appear to be irritated by particular food, as the skin is known to be by particular coverings; and this is not disease but idiosyncrasy. If, on the contrary, an individual can digest beef or mutton well, but suffers from heartburn, and other unpleasant feelings in the stomach when he eats pork, veal, or fried meat, the right conclusion is that his general powers of digestion are feeble, and easily depressed; and that his stomach is unable to convert into healthy chyme those aliments which require a higher degree of exertion. An healthy stomach, like every other healthy organ, possesses the power of preventing the chemical changes to which its contents would, under different circumstances, be exposed. Hence the food does not ferment there; but if the vital powers of that organ fail, the chemical affinities gain the ascendency, and, after a certain interval, various symptoms arise, which clearly denote the change that has occurred. This is the philosophy of an ordinary attack of indigestion. An uneasiness, and sensation of weight and distension are experienced in the region of the stomach, acidity prevails, eructations of disengaged air distress the patient, and nausea is felt from an effort of the stomach to eject its indigestible contents. There are chilliness, and a general lassitude, which originate from the sympathy that is produced on the nervous and sanguiferous systems. These effects particularly occur toward the end of chymification; and, in good time, pass off, while the remaining parts of the digestive process are apparently conducted with regularity. But this constitutes only a casual fit of dyspepsia, which may happen to healthy persons, and is remediable by clearing the bowels of any superfluous and crude matter, and avoiding the exciting causes in future. Yet, if an individual retire to rest with an overloaded stomach, he may pass into a comatose state, accompanied with apoplectic stertor, and from which it is not unfrequently difficult to rouse him. This condition springs from the sympathy of the brain with the oppressed stomach; and it is of great importance to distinguish such an affection, purely gastric, from genuine apoplexy, since, if the stomach be not relieved, the stupor will increase, and the sufferer perish. Such attacks are, however, not frequent, and principally occur when the muscular powers of the stomach are so impaired as to prevent the usual efforts which Nature makes to throw off an unmanageable burthen.

Should indigestion continue to recur in the stomach, the paroxysm will assume a more troublesome character; its symptoms will increase in number and extent, and the mischief will speedily involve other functions: but it may be useful to examine the causes to which the origin of the disease is attributable. In every change which the aliment undergoes there is the combined operation of *mechanical* and *chemical* agents. When, therefore, the food is introduced into the stomach, it owes its conversion into chyme to such combined actions, viz. *the chemical power of the gastric juice,* and *the mechanical,* or, in other words, *the muscular movements of the stomach;* which, in vernacular language, may aptly be called *churning.* It is to the failure or imperfect operation of the one or the other of those necessary actions, that indigestion is to be assigned. However perfectly the gastric juice may be secreted, if the mass be not sufficiently *churned,* it cannot become proper chyme; and the most active motions of the stomach will not compensate for a deficiency in the alimentary solvent. It signifies very little whether the paucity of the gastric liquor be absolute or relative, since, in either case, an indigestion must follow. The quality and quantity of the gastric fluid may be influenced by causes immediately acting upon the stomach, or by those which affect it through the medium of sympathy. Under the first class may be noticed those that produce a direct influence upon the nerves of the stomach, and without whose healthy action no secreting surface can perform its functions with regularity. Among these, the injudicious ingestion of narcotic substances, or of alcohol, deserves a distinguished attention. The languor springing from inanition also brings on a "discontented state of the stomach;" and, under this state, the gastric juice is not secreted in a healthy manner. But all these causes yield in importance when compared with those which act through the medium of sympathy, namely, the various passions of the mind, &c. the very singular and notorious sympathy which prevails between the skin and the stomach; and also of the latter viscus with the urinary organs. We are now to consider the causes that may operate in depressing, or paralysing the muscular powers of the stomach, and by which causes the mechanical process, essential to chymification, is imperfectly performed. Of these, undue distension is perhaps the most common, and, at the same time, the most powerful. This may be proved, not only from ample observation on the stomach, but by the analogy of other cavities; and particularly when the bladder is over-distended with urine, or the rectum by fæces. If this observation be applied to the stomach, it is easy to comprehend why, in an over-distended state of that viscus, vomiting can scarcely be excited by the most violent emetic; and it is also comprehensible, upon the same principle, how greatly the muscular fibres may become *permanently* debilitated by the repetition of such an excess. This over-distension is principally ascribable to food which has a tendency to swell from the heat and moisture of the stomach; and, from this circumstance, a person may eat without any sensation of fulness, and yet in the space of an hour he will experience it in a most distressing degree. This generally happens where much new bread has been eaten, or nuts; or from soda water; or, indeed, from any beverage which contains fixed air. There are certain postures of the body, common to shoemakers, taylors, engravers, and clerks at the writing desk, which, by preventing the necessary egress of the contents of the stomach, favour an accumulation in its cavity. In this, and similar ways, indigestion is either caused or exasperated.

2. *Imperfect Digestion in the Duodenum.* Here the author introduces the opinions of his friend Dr. Yeats, and as these are well known we must refer to the work itself. How far the duodenum deserves to be denominated a "*second stomach*," we have already discussed;* and how far the sentiments expressed concerning its pathology, and the conclusions drawn from them, are sound and practical, or visionary and theoretical, let others determine. But it is our duty to call attention to the subjoined extract.

"In some cases (of duodenal indigestion) a severe pain is felt in the back, especially in the region of the right kidney; and Dr. Yeats states a symptom, which I have also noticed on such occasions—a faint and fluttering pulse, occasioned by the pressure of the vena cava against the spine by the distended intestine." 226.

Is it come to this?—*A faint and fluttering pulse, in duodenal indigestion, is occasioned by the pressure of the vena cava against the spine by the distended intestine!* Is an assertion like this to be tolerated by a profession boasting, and justly boasting, of extended views, accurate observation, and rigid induction from proven facts? What is it calculated for but to deceive the unsuspecting, and to mislead the ignorant? What is it but to supply the want of knowledge by bold conjectures only, and to pretend to a perspicacity which is beyond humanity? Be it for ever borne in mind, and let it for ever

* New Series, No. 8, April 1826, p. 422.

stand as a monument of mortal vanity, that the assertion, now so deservedly reprobated, is made with all the confidence of demonstration; yet is it undeniable that no man can determine such a point, or render it even plausible, by sophistry. We call upon Dr. Paris and Dr. Yeats to explain to the public how they know that "*a faint and fluttering pulse is occasioned by the pressure of the vena cava against the spine by the distended intestine;*" for we do most unequivocally declare that the alleged circumstance is physically impossible from the anatomical construction of the parts. But admitting the possibility of such a pressure, what proof is there that it could produce "*a faint and fluttering pulse?*" as if such a pulse could not, and did not often, arise from various other, and more likely causes than a passive and polite vena cava permitting a rude and distended intestine to press it against the spine.*

3. *Of Head-aches which arise from Indigestion.* From the intimate sympathy which subsists between the nerves of the stomach and the brain, it is not extraordinary that any casual derangement of the digestive process should communicate its influence to the head. Dr. Warren† states, that there are two forms of dyspeptic head-aches; the one he refers to a fault in the stomach, the other to a defective action of the upper bowels. The former is distinguished by a languid and feeble, but not an unnaturally frequent pulse; the tongue is whitish and slightly coated; the edges are of a pale red colour. There exist a mistiness before the eyes, and general indistinctness of vision, also a dull pain or weight in the head, with some confusion, slight giddiness, and an apprehension of falling. These symptoms are attended with slight nausea, or an uneasiness and sense of irritation in the stomach; and often likewise by a

* Besides, we ask Dr. Paris and Dr. Yeats, in what possible way pressure on the cava inferior, which only *partly* supplies the *right* chambers of the heart, can cause a weak and fluttering pulse in the arteries arising from the *left* ventricle, after the blood has made the round of the short circulation in the lungs? In the whole course of our reading we never remember to have seen so strange a statement from any medical writer, or one so entirely untenable. If the heart of a man consisted of a single ventricle, and the distended duodenum could mechanically interrupt the flow of blood into that ventricle, there might be some colour for such an assertion. But such is not the case. Every man of clinical observation, and especially every man who has ever experienced dyspepsia, well knows, how much the action of the heart is influenced by any source of irritation in the stomach, duodenum, small intestines, and even the colon, without having recourse to such a clumsy mechanical explanation as pressure on the vena cava.—*Rev.*

† Medical Transactions, vol. iv. p. 233.

feeling of constriction about the fauces, accompanied with a watery secretion. Coldness and slight stiffness, or numbness of the fingers are sometimes present. The other parts of the system are, in general, affected with a great degree of nervous sensibility. The second species of headach, or that depending upon irritation of the bowels, probably in the duodenum, is remarkable for the appearance of brilliant ocular spectra, which distress the patient; there is chilliness of the body, and coldness and dampness of the hands and feet. The pain in the head is very severe, attended with a sensation of coldness and tightness of the scalp, slight giddiness, weight, distension, and stiffness of the eye-balls. In some cases, as these symptoms increase, they are accompanied by tingling and numbness of the fingers and hand. The tongue is commonly covered with a yellowish-white fur, and is often very considerably coated with it. The pulse is of the natural frequency, but languid; nausea is oftentimes present, yet seldom amounting to vomiting. There are usually flatulency, and a seeming dryness and inactivity of the bowels. This last symptom is pathognomonic; the sufferer feels as if his intestines had lost their sensibility, and were unable to propel their contents, which occasions a peculiar sensation of weight and obstruction. The appearance of the alvine discharges varies very much, yet they are always of an unhealthy kind. Generally, bile shews itself in too large quantity; sometimes of various colours, and of different degrees of viscidity; occasionally the evacuations have a natural look, but display portions of undigested food. At other times, the stools are of a faint yellow colour; and, floating upon water, they give an odour like that of saliva. A very common appearance, especially where there has been great dejection of spirits, is a loose discharge, of a dark greenish-brown colour, which smells like the grounds of sour beer.

The *stomach* headach generally occurs in the earlier stage of digestion;—that which may be termed the *duodenal** takes place when the food has passed into the intestine. The former

* In our opinion this is purely conjectural. How is it possible for Dr. Paris to know, except by mere supposition, that there is a *duodenal* headach, which, with singular good breeding, appears after the food has entered into the duodenum, lest it might previously have spoiled the appetite and interfered with chymification. Alas! diseases are not imbued with politeness; and it is apparently impossible for any physician to pronounce, with confidence, as this author does, that one headach occurs "*in the earlier stage of digestion,*" and the other arises "*when the food has passed into the duodenum;*" for, as we shall attempt to prove in the next note, such headachs most generally develop themselves when the stomach is, and has long been empty, and in a state of atony.—*Rev.*

is relieved by an emetic;—not so the latter, but a purgative mostly curtails it, because the offending cause is thereby expelled.

From the symptoms above related, a discrimination can be made between these two species of headachs. But pain in the head may arise from causes distinct from the alimentary canal; as from congestion of the brain; its internal disorganization; diseased bones of the skull; or from a deranged state of the nervous system. For the diagnostic symptoms, which are not at all ambiguous, we refer to the paper of Dr. Warren, or the pages of Dr. Paris.

The nervous headach is distinguished by the absence of constitutional disorder, and by the smallness of the space on the surface of the head which the pain occupies. There sometimes occurs a soreness of the scalp, with shooting pains, that are produced by the slightest touch; and these are probably dependent upon some biliary derangement. There is also a species of headach which would appear to depend upon a languid circulation through the brain;—this arises after an excess of wine; or, in females, during the catamenial discharge. It resembles numbness rather than pain, or that sensation producible by intense cold. The characteristic symptoms are, languor of the circulation, pallor of the countenance, and other indications of debility.

If the dyspeptic headach be allowed to take its course, it will generally terminate in a few hours; but, when it has become habitual, it is often protracted through one, two, or more days. It is cured by rectifying the errors of the digestive organs.*

---

* Without impeaching the accuracy of Dr. Warren's opinions on the stomachic headach, we shall briefly express our own. Long experience has convinced us that this affection most generally arises when the stomach is empty and in a state of atony. Hence, (and observation verifies the remark) it commonly invades early in the morning and in the evening;—periods when, the digestive process having been for hours completed, the stomach must necessarily be vacant and weak from its previous labours. The manner in which a cure, or, at worst, relief, occurs, confirms the correctness of these sentiments. Who has not seen a dyspeptic sufferer recline his aching head, early in the morning, either on the table or his hand, and be unwilling to speak? Yet, no sooner does he take breakfast than the headach usually departs, and he begins to converse, and even to be cheerful! Who has not noticed such a headach to be cured by dining, or by drinking a glass of wine, or of malt liquor? And, finally, who has not observed such a headach, occurring in the evening, and exceedingly distressing, to be dissipated by a light supper or a draught of wine? Every medical man must have witnessed these facts. The principle is the same in all, and in some others presently to be mentioned. The stomach is weak and void, the head sympathises with it, and

4. *Indigestion from Biliary Derangement.* Omitting this subject, which is done full justice to, but which contains nothing original, we advance to an infinitely more engaging one, namely,

5. *The Progress and Symptoms of Chronic Indigestion.* From considering a fit of indigestion in the stomach or duodenum, let us now proceed, says Dr. Paris, to whom, at present, we are historians, and not critics, to trace its consequences, when it is frequently repeated or protracted. In this case, other organs become successively involved, and a train of distressing and complicated symptoms arises. The dyspeptic patient having, for some time, suffered from those feelings of uneasiness which have already been described, experiences some diminution of his strength. This, at first, is only occasional, but growing worse, in spite of his best hopes, he now consults a physician. At this juncture, it is of great impor-

---

the exciting causes are banished by food, drink, or both. The seat of this headach is, apparently, in the occipito-frontalis muscle. The pain is singularly fugitive; changing its place repeatedly during two or three hours;—Sometimes over the frontal sinuses; then the forehead; next the temples, or occiput; and again to the first parts:—Occasionally occupying a very small, or large extent. This headach is, by many, called rheumatic, for the same texture is involved as in rheumatism; and every acute observer knows how intimately general rheumatism is connected with a disordered stomach and bowels. It is also, at times, termed a sick one, but the expression is objectionable, and calculated to mislead, since it seems to imply that the aching and sickness are co-eval, whereas it is the intensity of the pain that induces sickness, and which most readily arises in a weak and empty stomach. When the patient is able to vomit, though scarcely any thing is ejected, for obvious reasons, he is mostly cured of his headach, and why? Because the act of vomiting is a stimulus, tantamount to aliment, whether solid or liquid. Again, no person suffers from this headach while he endures pain in the stomach; for, when the pain commences, the headach vanishes almost instantaneously;—and wherefore? Because the pain is a stimulus, and alters that state of the stomach on which the headach depended. Thus is it clear, that the stomachic headach most generally springs from an atonous and vacant condition of the stomach; that such a headach is very commonly removed by stimulants; and that, of those particular stimulants, there are four kinds, namely, *food, drink, vomiting,* and *pain*—all of which operate upon a like principle, the overcoming of a state of atony in the stomach.

It will probably be objected that pain and vomiting ought rather to be classed as sedatives than as stimulants. Let any one observe the state of the circulation, the secretions, &c. during and after vomiting, and he will be convinced that this process is a general stimulus or excitement, while it lasts. The same may be said of pain. It quickens the pulse, and excites the whole system. But, waiving all argumentation about the terms stimulant and sedative, we maintain that the action of vomiting generally relieves the head—and that the return of pain in the stomach is as generally accompanied by a cessation of headach.—*Rev.*

tance to distinguish between a feeling of transient depression, invariably associated with alimentary disturbance, and that debility which announces a general diminution of constitutional energy. In the former case, there are periods in which the patient feels perfectly well and strong; while, in the latter, although his spirits may vary, he never rises to the healthy standard of vigour. His usual avocations are irksome, and even laborious; the slightest exercise occasions fatigue, and deluges him with sweat. The tongue is usually coated on the posterior part, and on its centre, with a brownish-yellow fur. The bowels are, by turns, constipated, and too much relaxed; the pulse, at this time, is generally slow and small, though somewhat hard; the countenance is more pallid than common; the eyes seem sunken, and their lids swollen, while the eye-balls are occasionally injected with yellow streaks. In some instances, heart-burn and a sense of oppression are experienced after meals; but, in others, only languor and extreme listlessness. On other occasions, a sense of constriction, is felt about the fauces, and a difficulty of swallowing, also, as if the œsophagus presented a mechanical obstruction to the transit of aliments. Dizziness; unusual drowsiness; pains in the head; ringing in the ears; a disagreeable taste in the mouth; an altered state of the salivary secretion, sometimes limpid, like water, and, at others, thick and ropy; palpitation and a sense of faintness are symptoms which, also, in a greater or less degree, commonly distress the dyspeptic sufferer. His hands are alternately hot and cold; in the former state they are dry, in the latter, more usually damp. His nights are sometimes, but not generally, disturbed by restlessness and uneasy dreams. He wakes in the morning without that feeling of refreshment which follows repose in health; is unwilling to rise, or, indeed, to move, for his limbs ache, the muscles of the trunk are even sore to the touch, and every change of position has its attendant inconvenience. Any alteration in the weather is felt as a serious evil;—if it become a degree or two colder, he creeps to the fire, and sarcastically and bitterly inveighs against the variableness of the climate; while, if its temperature be raised, he is oppressed with heat. His bowels become more and more untractable; the usual purgative ceases to produce its accustomed effect; he increases the dose, and, when it does operate, the action is too powerful, and its consequences are not easily checked;—a diarrhœa is established; and this again, in its turn, is superseded by still more obstinate constipation. This evil arises from the inconstant and unsettled state of the alimentary secretions, and it is not easy to graduate an artificial stimulant so that it shall always correspond with the varying state of the organs upon which it is to operate. The depression of the patient's spirits increases as the disease advances; he loses all hope; wastes in flesh, suffers a thousand distressing sensations, and fancies the existence of a thousand more. Wandering pains are felt in the bowels and side; a tenderness in the epigastrium is experienced on pressure; the abdomen is often preternaturally tense; his breathing is occasionally oppressed; a short dry cough distresses him, and expectoration is extremely difficult. If, at this period, the alvine discharges be inspected, they may present every variety of morbid appearance, since they may be unnatural in colour, odour, consistence, figure, or quantity; or yeasty, bilious, fatty, like tar, &c. &c. A diminished flow of deep-coloured urine is invariably associated with febrile action; while an increased quantity of pale urine more generally indicates a peculiar state of nervous irritability, without fever. But, in estimating this circumstance, we must inquire into the various excretions of the skin. *Albuminous* urine, in which a coagulum is produced by heat, is occasionally met with in dyspepsia; and Dr. Prout thinks that it is derived from the chyle. But of all changes in the urine, none, perhaps, are less equivocal, or more indicative of a deranged state of the alimentary functions, than the deposit of lithic acid, either in an amorphous or crystalline form. Sometimes the eating of bread induces such a change; and this lithic diathesis has been overcome by the substitution of biscuits. In this, and similar instances, the importance of judicious diet is astonishing. According to Dr. Prout, there are three divisions of lithic sediments, viz.—1. *Yellowish or nut-brown*; 2. *Reddish brown or lateritious; and*, 3. *Pink* sediment. The patient, to return from the digression we have made concerning urine, will, in this stage, sometimes labour under sudden disturbance on first falling asleep; fearful startings; catching of the limbs; thoracic distress, not unlike *angina pectoris*; together with imaginary flashes of fire, like lightning; and a numbness of the hands, which is, at times, only felt in one or two fingers. Usually there is sore throat, occasioned by relaxation; the skin is frequently dry, and even scaly; the tongue also becomes drier, and sometimes clean, and of a bright colour. Thus harrassed, the unhappy invalid, in vain, tries change of air: his emaciation increases; his ancles swell; and the general debility calls other diseases into action, various in kind and locality; but, commonly, the weakest part suffers. And "*when any new disease is permanently established, the original symptoms are mitigated, and sometimes wholly suspended; whereas, if*

*the new affection be only symptomatic, instead of relieving, it often aggravates them.*" The permanent tenderness of the epigastrium, and a clean, bright tongue, are the best indications of approaching mischief; and not hardness of the pulse, since it is very treacherous, and now and then soft and undulating, even under certain organic affections. Dr. Philip has *proved*, by his experience and reasoning, that "*long-continued irritation at length terminates in inflammation and organic derangement in the part affected.*"*

After indigestion has long harrassed the stomach, its villous coat becomes affected, and as the pylorus, from the peculiar nature of its office, is singularly exposed to the continued source of irritation, it is certainly very liable to inflammation, and this may, perhaps, explain the accession of the tenderness in the epigastrium. Yet here much doubt prevails, since the same often arises from various obvious and internal causes, and, hence, considerable caution is demanded, whether in opinion or practice. For, notwithstanding all Dr. Philip urges, it is

* We would be glad to learn from Dr. Paris where and how Dr. Philip has *proved* this position. We do not deny that sympathetic disorder (from dyspepsia) of a distant organ, as the heart, the brain, the kidneys, &c. may terminate in organic disease; but we protest against that *frequency* of such termination, which is every where to be *inferred*† from Dr. Philip's work. Thus, we will take head-ach, which is, in a given instance, to be considered as merely symptomatic of indigestion, the latter being (for the sake of argument) unequivocally present at the time the head-ach first appears. After a certain period, the patient dies, and, on dissection, we find organic disease of the brain, and no appearance of disease in the digestive organs. Here, then, Dr. Philip and Dr. Paris tell us, is a proof that the symptomatic disease has turned into organic, and leaves no trace in the original seat of disorder, the stomach. But we have just as good foundation for the opposite reasoning. We all know that the sympathy between the two organs is reciprocal. The affection of the head may be primary, and that of the stomach secondary. The disease, in the former, may go on to disorganization, and cause death, which is, in fact, often the case, and then, on dissection, nothing can be found wrong in the stomach. There is something like proof of this view of the case, but there is no such thing as a shadow of proof, but mere conjecture, in favour of the other. Here, then, is one of the many instances in which Dr. Paris has taken for granted the dicta of others, without giving himself the trouble to think for himself.

If Dr. Philip and Dr. Paris ask how it is that, as the disease becomes aggravated, or, in other words, structural in the brain, (allowing this to be the primary seat) the affection of the stomach subsides? We reply that they have answered this question themselves. They say that, while the head-affection is merely symptomatic, no relief is given to the stomach; but, when it becomes permanent, or organic, the stomach affection subsides. The explanation, therefore, bears either way. Dr. Johnson has fully taken up this subject in his new edition of the Influence of Tropical Climates, to which we refer

† We say *inferred*, rather than distinctly asserted.

difficult to imagine how serious mischief can take place in the pylorus without the occurrence of vomiting. When the bowels have long been disordered, their villous covering is tumid, turgid with blood, and sometimes ulcerated; and these appearances, according to Mr. Abernethy, are most manifest in the large intestines. Protracted dyspepsia likewise occasionally terminates in a disease of the mesenteric glands; and when the connexion which subsists between the function of the kidneys and that of the chylopoietic organs is considered, the disturbed appearance of the urine, and the supervention of calculous disorders, can excite no surprise in cases of dyspepsia.

Having finished this part of his subject, Dr. Paris presents the reader with a tabular arrangement, or scheme for investigating the causes, nature, and seat of indigestion; comprising leading questions concerning specific and general symptoms; particular circumstances; occasional questions; general observations upon physical character; and on collateral circumstances with respect to the season of the year, nature of prevailing epidemics, and the weather; and, finally, he comments on the relative importance of each with shrewdness and good sense. But with these parts we cannot meddle, since we must hasten forward to therapeutical matters.

### *Of the Cure of Indigestion, as it relates to Diet, Exercise, and Medicinal Treatment.*

The previous habits of the patient, and the origin and seat of the disorder, are the circumstances from which the physician is to derive his indications of cure. If the disease have not extended its influence beyond the stomach and bowels, the curative means will be more simple, and, at the same time, more prompt in their salutary operation, than where it has involved the functions of remote organs. But, in the latter instance, the symptoms are so frequently those of mere sympathy, that the practitioner has often the satisfaction of observing their removal by remedies that can only have acted on the primæ viæ.

"The dyspeptic patient should rise from his bed as soon as he wakes in the morning: for, as Mr. Abernethy justly states, 'many persons, upon first waking, feel alert, and disposed to rise, when, upon taking a second sleep, they become lethargic, can scarcely be awakened, and feel oppressed and indisposed to exercise for some time after they have risen.'*

* We can state, from pretty extensive observation of this class of disorders, that early rising, where a contrary habit has been long indulged in, is not

He should then walk, or rather saunter, for some time in the open air, previous to taking his breakfast, the material of which is to be selected according to the principles already discussed. He is now in a condition to follow his usual avocations; but it is a circumstance of no slight importance to procure an evacuation at this period, which is easily effected by habit; a person who accustoms himself to the act at a certain hour of the day, will generally (?) feel an inclination at the appointed season. The invalid should not allow his occupations, if sedentary, to engage him for more than three hours, after which, exercise on horseback, or by walking, should be uniformly taken." 262.

The hour of dinner should not be later than three o'clock, and the patient ought to enjoy an hour of previous rest. The dinner should consist of few articles; they must be carefully masticated; and the invalid should leave the table at the moment he perceives that the relish given by the appetite ceases.* The allowance of wine must be guided by former habits and present condition. It is essential that the patient should enjoy quietude for at least two hours after dinner. Afterwards he may walk or saunter for a short period; and at six or seven o'clock he may take tea, or some other diluting liquid; after which, exercise will be highly useful to assist the sanguification of the previous meal. In the summer season, active exertions, according to the sufferer's strength, will be advantageous. At ten o'clock he may take some toasted bread, or a lightly-boiled egg; and at eleven he may go to bed; and, if necessary, from previous custom, he may indulge himself with a glass of wine and water. The bed-room should be well ventilated, and its temperature ought, as nearly as possible, to be that of the apartment from which the invalid has retired. A well-stuffed mattress is to be preferred to a bed of down, and the curtains should not exclude the free circulation of air. He ought not to retire with cold feet, since nothing contributes more readily to disturbed sleep and uneasy dreams than the unequal circulation which takes place on such occasions.

---

very easily, or very safely, adopted all at once. In many instances where we have seen it tried, the patients have observed, that "they were fit for nothing all the day afterwards." It must, therefore, be cautiously commenced, though we acknowledge that, when the habit is once acquired, it is a powerful mean of preserving health.—*Rev.*

* Before this, in many cases. In a considerable proportion of dyspeptics, the appetite outstrips the digestion, and the grand criterion of the quantity is not the cessation of relish, but the state of the feelings one, two, or three hours after dinner. For the truth of this remark we appeal to the dyspeptic. We do not believe Dr. Paris ever laboured under indigestion himself.—*Rev.*

*Acidity of the Stomach, Flatulence, &c.*

In the opinion of our intelligent author, the acidity which occurs in the stomach of dyspeptic sufferers may, occasionally, depend upon the fermentation of the food, or a vitiated state of the gastric secretion. In cases of imperfect chymification, it would appear more generally to depend upon the matter generated by the food; for it is instantly relieved by a dose of carbonate of soda; but, where it is symptomatic of some disease in a distant organ, as the uterus, for instance, it would seem to be connected with an acid state of the gastric juice, and is, in consequence, not relieved by alkalies.

"I am not aware that this distinction has ever been established, but I am so well satisfied of its truth, that I have, in several cases, been led, from this circumstance alone, to distinguish between primary and symptomatic affections of the stomach. Unrelenting and continued cardialgia ought to lead us to suspect disease in some other organ; although I am not prepared to state that it is never the effect of a primary affection of the stomach."* 264.

In ordinary cases of acidity the bowels are to be opened, and the digestive organs brought into healthy action by a well regulated regimen, and by such medicines as may give tone to the stomach, and increase that propulsatory power of the intestines, which they demand to pass off the undigested portions of food. Purgatives are better than emetics, and still more so because the disease is generally complicated with fæcal congestions in the colon. Five grains of the *extr. colocynth. comp.* or of the *pil. cambog. comp.* combined with two of calomel, afford a very efficient remedy. This done, the peristaltic motion of the bowels may be kept in a state of gentle excitement by the following draught.

"℞. Sodæ Tartar.

—— Carbon. āā ℈ij.

Aquæ Anethi, f ℥i.

Tinct. Calumbæ, f ʒi. Fiat haust. cum acidi tartarici granis quindecem in aquæ semi-fluid-uncia solutis, in impetu effervescentiæ sumendus."

---

* We are convinced that obstinate cardialgia and acidity are, in nine cases out of ten, the consequence of eating too much in *quantity*, whatever that quantity may be. We have seen these complaints quickly cured by reducing one half of the food at dinner—confining the *quality* entirely to plain meat and stale bread, and permitting no other drink than a table-spoonful of brandy to a tumbler of water. Let this be tried, with half a grain or a grain of the sulphate of quinine thrice a day, and mark the effects. We, of course, allude to cases unconnected with organic disease of the pylorus. Where there is any suspicion of the latter, leeches, counter-irritation, and farinaceous food offer the only chance of arresting the progress of the malady.

The exhibition of alkalies and absorbent earths will remove the present evil, but not produce any beneficial influence in averting the cause. Carbonate of ammonia is, perhaps, the most efficacious of the antacids, for it neutralizes a portion of the acid matter which seems to exist in a gazeous state in the stomach, and, consequently, eludes the action of the fixed salts. It is, also, calculated to relieve the debility that is so common an attendant. After the bowels have been thoroughly evacuated, very small doses of opium are sometimes useful, especially when painful cardialgia exists, and which symptom is a common one of hepatic obstruction. In symptomatic affections, nitric acid has been serviceable. Diarrhœa not unusually arises from the irritation of the acrid matter, and for this, small doses of magnesia, with a few drops of laudanum, in the form of a draught, will prove generally beneficial. But permanent relief is to be sought in a change of food. All vegetables should be superseded by a diet of animal food; provided the stomach will consent, since that organ will never digest that against which the inclination rebels. Experience has demonstrated the pernicious effects of all fried articles, butter and greasy viands, pasty and crude vegetables, and likewise of astringent wines. Broths of every description, but principally those made of the meat of young animals, are a fruitful source of heart-burn. Veal contains a saccharine principle which is very susceptible of acetification. To invigorate the alimentary canal, the best medicines are those composed of pure vegetable bitters, with some aperient salt. The infusion of quassia, in the dose of half an ounce, with two drachms of the infusion of senna, and a drachm of tartrate of potass, warmed with any aromatic tincture, is an excellent compound. The infusion of rhubarb, quickened with a small proportion of the compound tincture of aloes, is a very useful remedy.

Flatulence is often a very distressing disease. Sometimes it is associated with acidity, but frequently it is the only symptom indicative of an imperfect digestion. Whether the gas, with which the bowels are inflated, be a product of fermentation, or a secretion by their vessels, is a question that has been much discussed. It may, probably, arise from either of these causes, although it is generally attributed to the first. In some cases, there is contained sulphuretted hydrogen gas, and eructations take place which are characterized by the smell of rotten eggs. Where this occurs, the existence of great alimentary disturbance may be inferred; since the natural affinities, by which the digestive changes are produced, appear to be subverted, and a new chain of compositions and decompositions established. The albumen would seem to be the substance from which this compound is generated; and in several instances relief has been obtained, by confining the patients to a strictly farinaceous diet. Ordinary cases of flatus are, however, of a different nature; the air seems to be the product of fermentation, and the cure consists in avoiding such vegetables as are of a flatulent kind. But an undue sensibility or irritability of the intestines may occasion distention without any morbid increase of gas, and particularly so in certain states of the alimentary canal. Hence, in such cases, we shall gain more advantage from calming the irritability of the bowels, than by attempting to dispel the flatus by carminatives. Small doses of the extract of hyosciamus, combined with two grains of ipecacuan, give relief in attacks of flatulence which have resisted common medicines.

Protracted dyspepsia not unfrequently depends upon a morbid condition of the alimentary surfaces. The mucous membrane becomes affected, and the disorder is not removed until the adoption of measures to restore its healthy secretions. The administration of a lenient purge every morning, with small doses of the *vinum colchici* repeated twice a day, is eminently successful. Where the dyspeptic disease is connected with duodenal irritation, no medicine is more useful than this wine, which is to be accompanied by occasional laxatives. Dr. Yeats highly commends the sulphate of potass—a scruple twice a day, in the infusion of quassia, and three grains of the *pil. hydrarg.* with or without two grains of extract of aloes, according to the state of the bowels. *Nothing is more mischievous than active purgation in every stage of dyspepsia.* This is a most important truth, and deserves to be recorded in italics. In cases even of loaded bowels, a gentle but continued stimulant is best, because less irritating; and when the biliary discharges are faulty, the *hydrarg. cum creta* and *pulv. ipecac.* are beneficial in doses of three or four grains. An infusion of senna, graduated in strength according to particular circumstances, and combined with small doses of some neutral salt, is the best form of a purgative. For accumulations in the colon a dose of the *pilul. cambog. comp.* is an effectual remedy. The *decoctum aloes compositum*, occasionally assisted by the *infus. armorac. comp.* is a truly valuable medicine in all affections connected with a torpid or irregular action of the muscular coats of the intestines. In such cases there are often spasm, distressing symptoms of flatus, borborigmi, eructations from the stomach; and the remedies most likely to afford relief are aloetic stimulants. The exhibition of the white mustard seed is very serviceable in cases which are marked by alimentary torpor; and in affections attended with muscular irritability; or in those associated with a diseased state of the mucous surfaces it is unquestionably useful. This seed is also advantageous in costive habits; and has been found to correct that species of diarrhœa which attends a diseased condition of the intestinal mucous membrane.

"The practitioner is well acquainted with the doctrines of Broussais: he maintains, that almost every disease arises from an inflammatory affection of the digestive canal; and, although the absurdity of so general a proposition must be admitted, we shall act wisely in suspecting the existence of such a state of disease in protracted dyspepsia: tenderness upon pressure, and the appearance of the discharges from the bowels, will generally announce such a condition; and lenient purges, the application of leeches, and a low diet, will furnish the best methods of treatment."—273.

The utility of bitters in certain states of dyspepsia is very questionable; and their exhibition is often a dangerous practice. Yet, where the disease springs from a mere want of tone, and is not complicated with intestinal irritation, they are, of all others, the most effectual medicines. Blisters are of eminent service in cases of irritation of the bowels, accompanied with tenderness on pressure; and they will frequently stop obstinate vomiting when other means have failed. The external application of heat to the region of the stomach will often allay gastric irritation that depends upon the presence of indigestible matter. The process of chymification is thus promoted by a species of contiguous and not very intelligible sympathy. It is the same when heat is applied to the feet, since this connexion is still less apparent, but that the digestion of a person in health may be arrested by the sudden access of cold to the lower extremities is a well-known and remarkable fact. The use of frictions, when applied to the abdominal region by a flesh-brush, deserves distinguished praise. The best time is night and morning, and the application should be continued for a few minutes.

We cannot do otherwise than omit the long and interesting observations on the use of flannels; the cold bath; practical directions for bathing; the warm bath; the shower bath; change of air; meteorological remarks; and change of scene and habits, which is as essential as the change of air.

For the unhappy individuals labouring under *tabes mesenterica*, the author, writing from experience only, and having uniformly found a vegetable diet to prove injurious, strongly recommends one entirely composed of animal matter. But to ensure a beneficial result, the meals must be scanty, and in quantity below what the appetite may demand; the intervals, also, between the repasts should be lengthened. In this way are the unwilling absorbents induced to perform their duties with greater promptitude and activity. In affections of this kind, the stomach rarely loses its powers; and it is less an object to provide easily digestible, than highly nutritive food.

Dr. Paris's observations on the diet of pulmonary invalids, and the treatment of phthisis are so brief, and, according to his account, which we see no reason to doubt, so important, that it would be unpardonable, if we declined to state them at full length, and in his own appropriate language.

"In tubercular affections of the lungs, it has been often disputed whether the low diet, so universally prescribed in such cases, is that which is best calculated to arrest the progress of the disease. From my residence at Penzance, and from the various cases which have fallen under my care, in consequence of having there practised, I may, without the risk of incurring the charge of presumption, assert, that few physicians have possessed greater opportunities of experience in this complaint than myself. The conclusions to which this has led me may be expressed in a very few words. Where there exists, in the earlier stages, great lassitude, coldness of the extremities, a quick but weak pulse, a tightness across the chest, as if it were confined by cords, but unaccompanied with acute pain in the side, a strictly vegetable diet is injurious. I have in such cases prescribed a regimen similar to that which I have above proposed for the cure of mesenteric affections; and I have certainly found it to be useful. To assert that I have cured an organic disease in the lungs would be more than foolish; but I have certainly arrested its progress in some cases, and I have restored others to perfect health, who had been gradually declining under a different treatment. Where a permanent cure has been effected, the presumption is, that the lungs were never actually deranged in structure; but the symptoms were of a nature to have justified such a conclusion. The medicine upon which I place the greatest reliance in such cases, is the extract of hemlock; and were I to express the extent of my own confidence in its powers, I might, perhaps, fall into the dangerous error, against which I have so strongly protested in my pharmacologia—that of bestowing such extravagant praise upon a remedy as to detract from its reputation. I shall, therefore, only observe, that this remedy tranquillizes the irritation of the lungs to a greater degree than any other medicine with which I am acquainted; but, in order to ensure so desirable an effect, it must be given in much larger doses than those in which it is usually administered. I usually commence with a dose of five grains, three times a day, which I gradually increase to ℈i, or even more: it will generally produce a slight giddiness, nausea, and a tremor of the body; a peculiar heavy sensation is also experienced about the eyes, and a tightness across the forehead; and the bowels frequently become relaxed: unless some of these sensations are produced, I consider that the remedy has not had a *fair trial.* The following is the formula for the preparation of the mixture which I have found to be so highly serviceable. If I am required to explain the *modus operandi* of each ingredient, I might, perhaps, fail in inspiring that confidence in its utility, to which I am convinced it is entitled. The practitioner must, therefore, rest satisfied with the results of experience, and accept facts in the place of theory.

"℞. Extract. Conii, et

Extract. Hyosciami, āā ℈ij.

Mucilaginis Acaciæ, f ʒij.

Tere simul, et adde

Liquoris Ammoniæ Acetatis, f ℥i.

Aquæ puræ, f ℥ivss.

Vini Ipecacuanhæ, f ʒi.

Syrupi Rhæados, f. ʒij.

Fiat mistura, de qua sumantur cochlearia duo ampla ter quotidie."—291.

After the above extract, there follows a history of five cases, illustrative of different states of dyspepsia, and with which the work terminates. We have striven hard to compress a large quantity of useful and various matter into a comparatively small space, but for this we claim no merit, since it is but right feeling in a public journalist not to presume upon what he has done, but only to feel regret that he has accomplished so little.

We close the pages of Dr. Paris's book with a favourable opinion of it, and of its ingenious author, and to recommend the one, and to eulogize the other, are acts no less of duty than of pleasure. The merits of the production are three-fold;—for it contains an excellent account of the anatomy, physiology, and pathology of the digestive, and collateral organs—presents a valuable treatise on alimentary matters—and exhibits a respectable dissertation on the stages of indigestion, and their modes of cure. We entertain no doubt that it will be particularly interesting, and highly acceptable, not only to the mere medical practitioner, and the dyspeptic invalid, but to the man of science, and to the inquisitive gentleman. For the sake of the latter class we could have wished the style had been more terse, correct, and polished, yet, where there is so much merit, it is scarcely fair to complain of trifling blemishes, whether of diction, or of matter. In him, who has written on almost every subject from the highest to the lowest, who has ably explained the nature of the coagulating, antiputrescent, and solvent powers of the gastric juice, and then stooped from the zenith of his eagle-flight to examine into the preparation of puddings and pancakes, needless repetitions, palpable solecisms, and stagnation of language may well be forgiven, and severe would be the mind, and frigid the heart, which could visit them with censure.

We had intended to express our own opinions on dyspepsia, but, having most unusually exceeded our limits, we are obliged to reserve them for a future opportunity; and shall only observe that, in our judgment, Dr. Paris has directed too little attention, indeed, hardly any, to the operation of mental causes in the production of that disorder, which are, according to our experience, more operative than all other causes united. That constitutional derangements, that excess, or deficiency of living, that inscrutable local circumstances, that hereditary pre-disposition may lay the foundation for, and ultimately excite indigestion, are truths which no observing man will question; yet these truths, confessedly influential as they are, sink into comparative insignificancy, when contrasted with the various and powerful affections of the mind, which exercise a harrassing and distressing dominion over the stomach, while this organ, disdaining to be an unresisting sufferer, re-acts at last, and commits reprisals in its turn. In every age and nation, general literature, as well as traditional story, attests the melancholy fact, that the sensitive, the highly gifted, and the most intellectual, have always been the victims to diseases of the head and stomach, and will continue to be until the human heart has ceased to beat.

---

It has been proved by a contemporary reviewer, that Dr. Paris has sometimes made use of the *scissors* in the composition of his treatise on diet, without the ceremony of acknowledgment. The former is excusable in a work like this—for a man must have lived half his life in a *kitchen*—indeed, he must be a complete *Kitchener*, to write, from personal observation, on the thousand compounds which enter into the human stomach, in the shape of food and drink. We do not wonder, therefore, that he has laid Sir John Sinclair under heavy contributions—that worthy baronet, who has ransacked earth, seas, and skies for materials to keep the frail tabernacle of man a few years longer than usual out of the hands of the undertaker. It certainly would have been wise in Dr. Paris, however, to have guarded his pages and paragraphs with those very significant little landmarks between *meum* and *tuum*, called inverted commas, and thus set the critic at defiance. This, by the way, is Dr. Paris's business, and doubtless he will look to it in a new edition.

Before we take leave of the work, we are induced, for several reasons, to notice one of the cases, the first on the list, of which the following is an abstract.

*Case.* A. B. (which, by the bye, we have reason to know, means Mr. P.) a gentleman of rank and fortune, aged 24, had suffered, for several months, from occasional head-ach in the evening, at first relieved by strong coffee; but gradually increasing in severity and with shorter intervals. It sometimes attacked him in the mornings, accompanied by sickness and the ejection of strong acid from the stomach, by which the paroxysm was terminated. He was athletic, florid, accustomed to strong exercise, had a good appetite, and ate accordingly. He was temperate in wine. He wrote to Dr. Paris in April, 1824, that his medical attendant was giving him aloes, blue pill, bitters, and alkalies, but that he derived more benefit from lime water than all the other medicines. He was fed on animal food and prohibited from vegetables. From this period he gradually grew worse—his head-achs increasing in severity, till relieved by the discharge of bile and acid from the stomach. He lost flesh, but still was not emaciated. Dr. P. prescribed the carbonate of ammonia an hour after dinner, and animal diet. "He was well purged, and the action of the bowels kept up by small doses of neutral salt. The stools were always natural in appearance." The slightest deviation from the prescribed diet produced head-ach. In November of the same year, he reported to Dr. P. that the perpetual recurrence of his old head-achs left him nothing for it but to turn them into ridicule. And so he did, in his narrative, but it turned out to be no joke in the end. By a steady perseverance, however, in his plan of diet and medicines, Dr. P. informs us, he found considerable alleviation, till March 1825, when the complaint returned as violent as ever, apparently from relaxation in dietetics. On the 18th May, he had a severe attack from the eating of a minced pie, and he complained from this time of listlessness and want of energy. He arrived in London in June, and Dr. P. found him much thinner, but better then than he expected. Here he had rather too many friends, and neglected his diet. He repaired to Leamington, and met with an accident, by which he suffered the amputation of a finger. His health is said to have declined "under this operation"—he lost flesh—experienced increase of head-achs—called in the aid of a popular practitioner (Mr. Jephson)—"under whose superintendance he took drastic doses of scammony, not only without relief, but with an *evident* aggravation of the symptoms." His bowels now (for the first time it is said) exhibited signs of torpor. Strong medicines soon lost their effect, and were followed by stronger, with the same result. It appears from his letter that he was, soon after this, in Ireland, where his medical attendant contrived "a more active combination," which he took daily, but still with decreasing effect. In December Dr. P. received a letter from him, which was nearly illegible, stating that he could scarcely see his own hand, not from dizziness, but from an indistinctness of vision, which became so great at last that he could no longer correspond with Dr. P. He repaired, therefore, to London; "but such was his weakness, and so severe his sickness, that he was many days on the road. *His face, hands, body, and legs, swelled to a considerable degree, and he experienced great difficulty in breathing.* A consultation was summoned, and, as he had had no motion for ten days, Dr. Paris directed ten drops of croton oil without effect, then a scruple of calomel with five grains of scammony, and a dose of the black draught. After some hours, the bowels answered, when a perfectly figured and healthy motion was the result. He was cupped, and blistered on the back of the neck, but his vision became daily more obscure.

His head-ach was relieved, but he constantly experienced a sense of weight and uneasiness in the region of the stomach. *His pulse was regular, but hard, and upwards of* 100 *in the minute.* In this state he continued for ten days, when he was seized with a violent dyspnœa, which was succeeded by hæmorrhage from the lungs, which terminated his existence; in spite of venesection and the usual means. What was the nature of the disease? "I confess that I had long considered the brain as its seat, and *I explained the dyspnœa from a deficient supply of nervous energy, his symptoms bearing a striking analogy to those which were produced by a division of the eighth pair of nerves.*"

"On opening the thorax, the lungs appeared so gorged with blood, as almost to resemble the spleen in texture; they were emphysematous in several places. The heart was apparently healthy in external appearance, but of a large size; upon making an opening into the right auricle and ventricle, these cavities were morbidly dilated, so as to constitute what has been termed *passive aneurism:* their parietes were not thickened. The left ventricle was also unusually large: the valves were in a healthy condition. Upon opening the head, the structure of the brain and its membranes were found in a perfectly healthy state, but without the usual presence of blood. The substance of the brain itself was perfectly blanched, and, upon cutting into it, the usual spots of blood were not produced. This organ, therefore, although not injured in structure, must have been unfitted for the performance of its functions from a deficiency of blood; in consequence, probably, of the feeble action of the heart. The history of this extraordinary case will admit of much physiological speculation. That the heart was the primary seat of the disease, appears to be the most probable conjecture; the loss of vision must have arisen from a deficient circulation through the brain; and to this also we are to attribute the obstinate state of the bowels. The derangements of the stomach may be referred to its sympathetic relations to the brain. The gorged state of the lungs may be accounted for, either by the imperfect action of the heart, or by the deficiency of nervous energy; for a similar appearance is observed in cases of narcotic poisoning, where the death of the animal takes place from the destruotion of the powers of the brain. I have lately met with a case of diseased heart, in which the patient complained of a similar imperfection in his vision; and he died in consequence of pulmonary hæmorrhage. I had no opportunity of examining the body." 300.

We think the history of this case will admit of more than "physiological speculation."

We observed that the instances of *symptomatic* affections changing into *organic* diseases, were not nearly so frequent as might be inferred from the writings of Dr. Philip. The foregoing case offers a remarkable illustration of this position. That the primary disease was that of the heart, is so obvious, that Dr. Paris himself, with commendable candour, admits it, at the expense of his previous prognosis. Yet the symptomatic *disorders* (for they do not properly deserve the name of *diseases*) of the head and stomach, were far more violent and distressing than the greater number of *organic* diseases of those parts usually are. The symptoms went on for years, and yet, on dissection, no alteration whatever of structure was appreciable in the brain or the stomach. Nothing can be more illustrative than this. Had this gentleman not been examined, *post mortem*, the followers of Dr. Philip would have said—"Oh, here was organic disease of the brain, induced by indigestion, as evinced by the intense pain, and ultimately by the privation of vision," &c. Happily dissection revealed the true nature of the disease.

Dr. Paris has denominated the above-mentioned condition of the heart "*passive aneurism;*" but in this we must beg leave to differ from him. Passive aneurism is dilatation of the chambers, with attenuation of the parietes—active aneurism is thickening of the parietes, with or without dilatation of the chambers. Dilatation of the chambers, without thickening or attenuation of the parietes, is properly termed *aneurism* or dilatation of the organ. "Enfin la troisieme forme, &c. distingue des precedentes, en ce que les parois dilatées conservent leur epaisseur naturelle."* This form of organic disease of the heart is by no means uncommon, and is, strictly speaking, an hypertrophy of the organ, since the whole volume is enlarged, without attenuation of its parietes. Now, it is well known that diseases of the brain, as apoplexy, &c. and affections of the respiratory apparatus, as asthma, are the common effects of these enlargements of the heart. Hence the long-continued head-aches—hence the difficulty of breathing, and—hence the final hæmorrhage from the lungs, and death of the patient. But were there no symptoms during life that might have led to the detection of the real nature of the disease? Was swelling of the face, hands, body, and legs, with "great difficulty of breathing," to be considered indicative of disease of the brain, or of "a deficient supply of nervous energy?" We appeal to every one who knows any thing of organic diseases of the heart, whether these symptoms are not the very surest signs of such diseases. As for the theory of defective nervous energy, and "division of the eighth pair of nerves," Dr. Paris cannot possibly be in earnest—it is evident that he is quizzing his friend Dr. W. Philip.

But we have now to ask, not Dr. Paris alone, but all the professional attendants, from Mr. Jephson up to the highest medical character consulted in this deplorable case, how and why it was, that the chest was never examined, and that "a gentleman of rank and fortune" was suffered to die of aneurism of the heart, without the slightest suspicion ever being entertained of the existence of such a malady? Here is another

* Traité des Maladies du Cœur, par M. Bertin et M. Bouillaud, 1824.

illustration of the neglect of the study of auscultation, still more remarkable than that which we recorded in our last number, page 606. This indisposition to a new study is rapidly working its own reformation. The rising generation of the profession are all making themselves acquainted with the immortal discovery of Laennec, and let those who despise such information, look to the consequences in a very few years.

Is it possible that Dr. Paris can be serious, when he attributes the blanched appearance of the brain, *in a man who died of hæmorrhage*, to deficient circulation in that organ during life, the patient having *aneurism of the heart*, with "a regular, hard pulse," above 100 in the minute?—It cannot be.

That the torpor of the bowels was dependent, in a great measure, on affection of the brain, in the above case, we are inclined to believe; but this affection of the head we consider to be just the reverse of what Dr. Paris believed it—so far from thinking there was "deficient circulation through the brain," before death, we are fully convinced there was a too active circulation there. How Dr. Paris could expect to find the brain very bloody after what took place in the lungs, we cannot imagine.

In stating and discussing this case, now put on public record, and consequently open to critical investigation, we have candidly and unequivocally differed from Dr. Paris, on several important pathological points, and the public will decide who is right and who is wrong. In conclusion, we can safely and conscientiously declare, that we have, in our analysis, been more on the watch for objects to praise than defects to censure—and that we have on all occasions adhered to the precept of the Roman satirist—

> "Quæ laudanda forent, et quæ culpanda, vicissim

> Illa *prius* creta,—*mox* hæc carbone notamus."

